# Molecular pathway of near-infrared laser phototoxicity involves ATF-4 orchestrated ER stress

**DOI:** 10.1038/srep10581

**Published:** 2015-06-01

**Authors:** Imran Khan, Elieza Tang, Praveen Arany

**Affiliations:** 1 Cell Regulation and Control Unit, National Institute of Dental and Craniofacial Research, National Institutes of Health, Bethesda MD.

## Abstract

High power lasers are used extensively in medicine while lower power applications are popular for optical imaging, optogenetics, skin rejuvenation and a therapeutic modality termed photobiomodulation (PBM). This study addresses the therapeutic dose limits, biological safety and molecular pathway of near-infrared (NIR) laser phototoxicity. Increased erythema and tissue damage were noted in mice skin and cytotoxicity in cell cultures at phototoxic laser doses involving generation of reactive oxygen species (ROS) coupled with a rise in surface temperature (>45 °C). NIR laser phototoxicity results from Activating Transcription Factor-4 (ATF-4) mediated endoplasmic reticulum stress and autophagy. Neutralizations of heat or ROS and overexpressing ATF-4 were noted to rescue NIR laser phototoxicity. Further, NIR laser mediated phototoxicity was noted to be non-genotoxic and non-mutagenic. This study outlines the mechanism of NIR laser phototoxicity and the utility of monitoring surface temperature and ATF4 expression as potential biomarkers to develop safe and effective clinical applications.

Light has a key role in human health as evident from its established roles in vision, vitamin-D metabolism, circadian rhythm and psychosocial state. Current popular clinical applications of light include Phototherapy (UV), Photodynamic therapy (PDT) and skin rejuvenation as well as high power surgical lasers in ophthalmology, dermatology and oncology^1^,^2^,^3^. Low power near-infrared (NIR) lasers are becoming increasingly popular for a wide range of biomedical applications including optical imaging, optical tweezers and optogenetics^4^,^5^,^6^. A less known therapeutic clinical application is the use of non-thermal, low power visible and near-infrared light to promote wound healing, hair growth, tissue regeneration or reduce pain and inflammation termed Low Level Light/Laser Therapy (LLLT) or Photobiomodulation (PBM)^7^,^8^,^9^,^10^,^11^,^12^,^13^,^14^. Despite their popular clinical usage, there is surprisingly little information on the biological responses and safety of near-infrared (NIR) lasers. Moreover, PBM therapy demonstrates a biphasic dose response (Arndt-Schulz) curve, where low doses appear to have beneficial therapeutic effects while higher doses are harmful (phototoxic), highlighting a critical role for determining precise therapeutic laser dose thresholds for clinical use^15^,^16^,^17^,^18^,^19^,^20^,^21^,^22^.

In this study, we observed that the NIR laser dose that results in detrimental effects (erythema and tissue damage) on mice skin correlates with a rise in surface temperature (≥45 °C). In a simulated, *in vitro* model where laser phototoxicity could be attained with high precision, the molecular mechanisms of NIR laser phototoxicity were assessed. Increasing laser doses generates heat and ROS damage that induced ER stress-mediated by Activation Transcription Factor 4 (ATF-4) and Heat Shock Protein 70 (HSP70) resulting in autophagy. However, when phototoxic damage is excessive and irreparable, detrimental effects were heralded by reduced ATF-4 and HSP70 levels and increased autophagy and apoptosis. Thus, these observations suggest that ensuring surface temperature <45 ^o^C during laser treatments, along with markers of ER stress specifically ATF-4, could predict maximal dose threshold for NIR laser applications.

## Results

### Surface temperature predicts laser phototoxicity in mice skin

**Figure 1:**
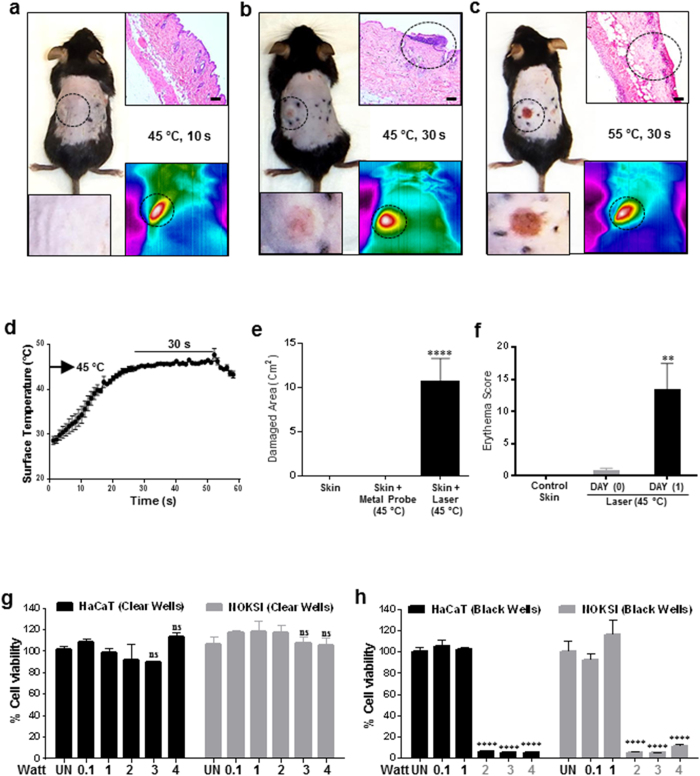
Laser induces phototoxicity *in vivo* and *in vitro*. Dorsal skin of 5-week-old C57BL/6NCr male mice were shaved and naired and were dynamically treated with 3.2 W laser to maintain (**a**) 45 °C for 10 s, (**b**) 45 °C for 30 s and (**c**) 55 °C for 30 s. The inset panels show (upper right) H&E staining and (lower right) skin surface temperature as monitored by the thermal camera and (lower left) higher magnification of clinical image. Scale bars = 70 μm (**d**) Surface temperature profile of mice treated with laser at 45 °C for 30 s as measured by the thermal camera (n = 13). (**e**) Damaged skin area was measured a day after laser treatment (45 °C for 30 s) and metal probe (n = 13 each). (**f**) Erythema scores measured immediately after treatment and 24 hours after phototoxic laser dose treatments. Significance based on one way ANOVA with the respective controls (n = 13). Laser treatments were performed in clear (**g**) and black (**h**) well plates on HaCaT and NOKSI cells and cellular viability was assessed 24 hrs after treatment with AlamarBlue as quantitated with a plate reader. Significance was determined using two-way analysis of variance (ANOVA) among different treatments using the Bonferroni’s multiple comparison test (n = 3). Statistical significance is denoted as *P* values <0.001 (**) and <0.00001 (****).

Several studies using low power lasers have reported detrimental phototoxic effects^23^. But parameters that could predict NIR laser phototoxicity precisely have not been reported. As higher laser doses are noted to increase tissue temperature, we first performed a dose escalation study in mice by varying irradiance (Watts/cm^2^) and time (s) and monitoring surface temperature concurrently. Laser phototoxicity was assessed using erythema score and damaged skin area at 24 hours post-treatment. We observed that laser treatment for 30 seconds with an increase in surface temperature to 45 °C were the most robust parameters for predicting laser phototoxicity (Fig. 1b,d–f and Supplementary Figs. 1a–f). Reducing treatment time (10 s) or increasing surface temperature (>55 °C) leads to absence of or excessive (epithelium, connective tissue and neuromuscular) phototoxicity respectively (Fig. 1a,c, Supplementary Figs. 1g–l, Supplementary Table 1 and Supplementary Video 1). Surface temperatures, along with treatment times, were chosen over irradiance and fluence (time), as latter vary tremendously with skin absorption (color) as evident from the correlation of melanin score, laser power and treatment time needed to attain surface temperature of 45 °C (Supplementary Fig. 2a–f). Phototoxicity induced by the laser was not merely thermal as a metal probe heated to 45 °C failed to induce significant skin damage (Fig. 1e and Supplementary Fig. 2g). These observations suggest that a safe laser dose can be used clinically by monitoring skin surface temperature during treatment which predicts phototoxicity despite variations in target tissue color.

### Laser phototoxicity is mediated by heat and ROS

To dissect the causal pathways of laser induced phototoxicity, *in vitro* experiments with HaCaT (human dermal keratinocytes) and NOKSI cells (Normal oral keratinocytes) were performed. We noted that laser treatments with increasing doses in clear plastic wells failed to induce significant phototoxicity (Fig. 1g). We had observed that the absorption of laser through mice skin depends on melanin score and about 50% to 98% of the incident light (810 nm laser) is absorbed by the skin of pale (mean melanin score 48) and dark (mean melanin score 98) colored mice respectively (Supplementary Fig. 2h). The remaining light appears to be absorbed by underlying tissues (muscles and bones). Based on these *in vivo* absorption patterns, an *in vitro* model was developed where laser treatments were performed on HaCaT and NOKSI cells seeded in clear (<10% absorption) and black (100% absorption) well plates (Supplementary Fig. 2h). We noted that laser phototoxicity was evident at doses of ≥27 J/cm^2^ (0.09 W/cm^2^; 2 W in 6 wells) in black well plates alone (Fig. 1h and Supplementary Fig. 2i). The percentages of well surfaces covered with black tape correlated with extent of cytotoxicity indicating that the amount of absorbed laser dose appeared to be critical (Supplementary Fig. 3a).

**Figure 2:**
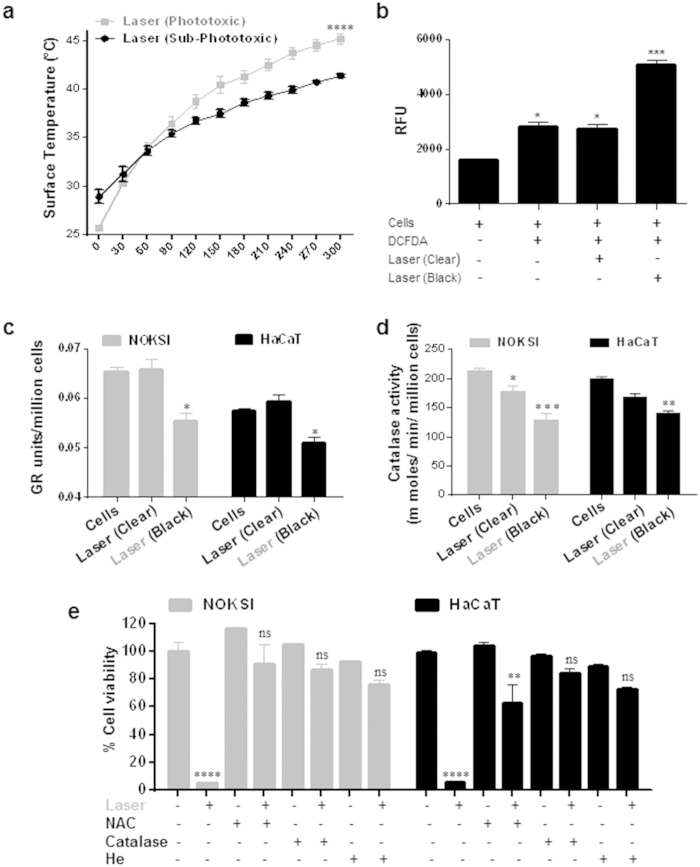
Laser induced phototoxicity is mediated by heat and ROS. Surface temperature of cells treated with laser as assessed by IR camera (n = 3) (**a**) is shown. (**b**) ROS generation was assessed with DCFDA by FACS analyses following laser treatments in clear and black well plates (n = 3). *In vitro* activity of GR (**c**) and Catalase (**d**) were assessed in HaCaT and NOKSI cells treated with phototoxic laser doses (n = 3). (**e**) HaCaT and NOKSI cells were pre incubated with NAC (1mM), Catalase (1000 units/ml) or Helium (bubbled for 5 minute) and treated with phototoxic laser dose followed by cell viability assays (n = 3). Significance was determined using two-way ANOVA and Bonferroni’s multiple comparison tests. Phototoxic laser doses are highlighted in gray font. Statistical significance are indicated as *P* *<* 0.05 (*), <0.001 (**), <0.0001 (***) and <0.00001(****).

Absorption of photonic energy leads to increased temperature and laser phototoxicity *in vitro* was also noted to correlate with a rise in surface temperature (≥45 °C) (Fig. 2a and Supplementary Fig. 3b). This rise in surface temperature appears to be critical for laser phototoxicity as treatments at 4 °C did not generate phototoxicity (Supplementary Fig. 3c). In contrast, pre-incubation of cells at 42 °C followed by laser treatment leads to significant phototoxicity at lower doses (1.5 W versus 2 W) (Supplementary Fig. 3d). For a given amount of energy, rise in temperature depends on mass and specific heat of the conducting medium. To further characterize our *in vitro* model, we noted that increasing mass (volume) or specific heat (PBS versus 10% serum DMEM) of culture media modulated phototoxicity significantly (Supplementary Fig. 3e,f). TUNEL assay demonstrated NIR laser cytotoxicity involves apoptosis (Supplementary Fig. 3g).

Absorption of photonic energy by molecular chromophores (photoabsorbers) results in a photochemical reaction leading to generation of ROS^24^. To evaluate the role of ROS in laser phototoxicity, ROS induction was assessed using a florescent probe (DCFDA). Laser treatments in black wells induced significantly higher level of ROS as compared to treatments in clear wells (Fig. 2b). Cellular redox status is determined by the presence of key ROS scavengers. Purified Glutathione Reductase (GR) and Catalase (CAT) enzymes were treated with phototoxic laser doses in both clear and black well plates and their activities were measured. It was observed that laser treatment on purified GR and CAT leads to significant reduction in their activity in black well plates selectively, without significant effects in clear wells (Fig. Supplementary 3h,i). We also assessed the cellular activity of GR and CAT enzymes in HaCaT and NOKSI cells. Laser treatment in both cell lines demonstrated a similar decreased GR and CAT activity selectively in black well plates (Fig. 2c,d). As the enzyme GR recycles GSSG (oxidized glutathione) to GSH (reduced glutathione) and helps in maintaining the reduced glutathione pool, phototoxic NIR laser treatments demonstrated significant increase in GSSG/GSH ratios (Supplementary Fig. 2j).

To ensure specificity of ROS mediated laser phototoxicity, pretreatment of cells with N-Acetyl-L-cysteine (NAC) and Catalase prior to laser treatment were performed which were noted to significantly rescue laser phototoxicity in both the cell lines (Fig. 2e). To further dissect the key laser phototoxicity process (Heat or ROS), rise in temperature was monitored during ROS neutralization experiments. We noted a similar rise in surface temperature in all ROS neutralization treatments that prevented cytotoxicity, suggesting ROS is a primary effector of laser phototoxicity (Supplementary Fig. 3k). To validate this further, replacement of Oxygen (necessary source for ROS generation) with Helium in the media resulted in alleviation of laser phototoxicity despite the noted increase in surface temperature (Fig. 2e and Supplementary Fig. 3k). These observations suggest that laser-generated heat (upstream) inactivates ROS scavengers that act along with dose-dependent ROS (effector) generation to result in phototoxic tissue damage.

### Laser treatments do not generate direct DNA damage

**Figure 3:**
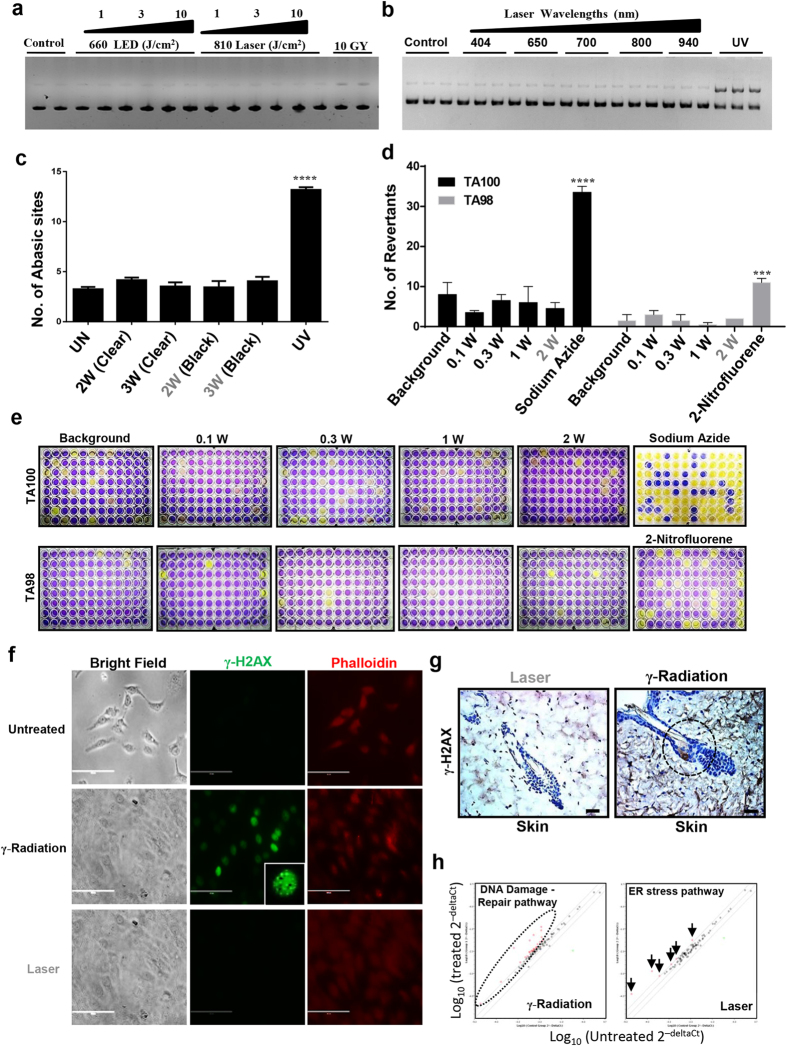
Laser does not cause DNA damage (Non-genotoxic). Plasmid cleavage assay was performed using PUC19 and following laser treatments with varying doses (**a**) and wavelengths (**b**) plasmid was analyzed by gel electrophoresis on 1% agarose gel that was quantitated by densitometry (n = 3). HaCaT (**c**) cells were treated with different doses of laser and genomic DNA was assayed for number of abasic sites. UV treatment was used as a positive control, Significance was based on one-way ANOVA with the respective controls (n = 3). (**d** and **e**) Ames test was performed on TA100 and TA98 strains of the *Salmonella typhimurium* using different doses of laser and revertants were quantitated on 5^th^ day after treatment. Significance was noted as per the manufacturer’s manual (n = 3). HaCaT cells (**f**) (Scale bars = 200 μm) or mice (**g**) were treated with phototoxic laser dose and γ-H2AX immunostaining was performed to assess DNA damage. γ-radiation (10 Gy) was used as positive control. Scale bars = 70 μm. (**h**) HaCaT cells were treated with radiation (10 Gy) and sub-phototoxic laser doses and PCR arrays were performed. Representative scatter plot of differentially regulated genes (fold change ≥ 2) are shown. Detailed list of genes are available in supporting materials (n = 2). Statistical significance are indicated as *P* *<* 0.0005 (***) and <0.00001(****).

From the above observations, a major clinical concern was if excessive ROS generation by laser treatments leads to direct DNA damage that could result in genotoxicity and potential mutagenicity. To address this, we first assessed the ability of laser to induce direct DNA damage using plasmid cleavage assay. Phototoxicity at higher doses with various light sources (broad-band light, laser and LEDs) along with doses used for PBM therapy did not induce DNA cleavage, in contrast to UV treatments (positive control) (Fig. 3a,b and Supplementary Fig. 4a-f). Direct damage by reactive oxygen species to DNA can lead to formation of abasic (apurinic, AP) sites during the process of base excision and repair of oxidized, deaminated and alkylated bases. Laser treatments at sub-phototoxic and phototoxic doses did not generate significant AP sites in both cell lines (HaCaT and NOKSI) in contrast to UV treatments (Fig. 3c and Supplementary Fig. 4g,h).

Sub-lethal DNA damage could potentially promote mutagenicity and cell transformation. To assess the potential of laser treatments to generate this form of genotoxic damage, Ames test was performed with two strains (TA100 and TA98) of *Salmonella typhimurium*. DNA damage in terms of changes in the frequency of revertants was assessed following increasing doses of laser treatments. None of the laser doses were noted to increase frequency of revertants above the background in both strains (Fig. 3d,e). A robustly validated biomarker of mammalian genotoxicity is γ-H2AX phosphorylation^25^,^26^. Treatment of cells and mice with phototoxic dose of laser does not induce phosphorylation of γ-H2AX *in vitro* or *in vivo* (Fig. 3f,g and Supplementary Fig. 4i). While high power lasers are capable of cytotoxicity, these results indicate that this does not appear to be mediated by direct DNA damage. This also implies that high NIR laser doses can be phototoxic without being genotoxic or mutagenic, indicating they can be safely used for clinical applications.

### Cellular responses mediating laser phototoxicity

**Figure 4:**
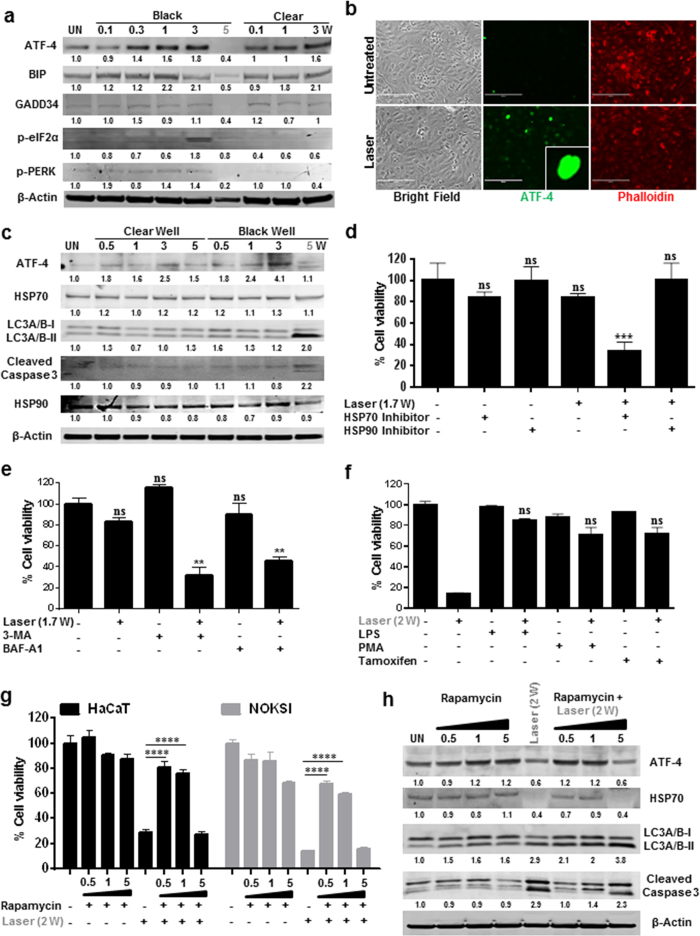
Laser induces ER stress at sub-phototoxic doses. (**a**) ER stress markers after varying doses of laser treatment were assessed at 24 hrs using immunoblotting. (**b**) Localization of ATF 4 in HaCaT cells were assessed after 6 hrs following laser treatment. Scale bars = 200 μm. (**c**) Level of autophagy (LC3A/B-ii/i), ATF-4 and HSP70 were assessed by immunoblotting after 24 hrs of laser treatment. HaCaT cells were pretreated with HSP inhibitors (**d**) autophagy inhibitors (**e**) or pre-treated with LPS (2 ng/ml), PMA (1X) and TMFC (Tamoxifen Citrate 40 μM) (**f**) for 2 hrs and were then challenged with laser treatment and cell viability was assessed at 24 hrs. Significance based on one-way ANOVA with the respective controls (n = 3). HaCaT and NOKSI cells were pretreated with varying concentrations of Rapamycin and challenged with phototoxic dose followed by the assessment of cellular viability (**g**) and immunoblotting (**h**) Images in a gel are cropped (horizontally) and placed together for better clarity of results and were run in the same experimental conditions. Significance was determined using two-way ANOVA among different treatments using the Bonferroni’s multiple comparison test. Statistical significance are indicated as *P* *<* 0.0005 (***), <0.00001(****) and not significant (n.s.).

To assess the molecular pathways mediating laser phototoxicity, we performed pathway specific PCR arrays. Treatment of HaCaT cells with ionizing gamma radiation robustly induces several genes (26 out of 90) known to be directly involved in DNA damage and repair pathways (Fig. 3h, Supplementary Fig. 5a and Supplementary Table 2 and 3). In contrast, laser treatment induced fewer genes that were all part of the endoplasmic reticulum (ER) stress pathway (Fig. 3h and Supplementary Fig. 5a). Therefore, it appeared likely that the laser phototoxicity may result from activation of ER stress pathway. Activation of the ER stress response occurs via three distinct receptors, inositol-requiring enzyme 1α (IRE1α), activating transcription factor 6 (ATF6) and protein kinase RNA-like ER kinase (PERK) leads to either repair (via refolding or autophagy) or cell death by apoptosis^27^. Phosphorylation of PERK leads to attenuation of general protein synthesis by the phosphorylation of eukaryotic translation initiation factor 2α (eIF2α) and selective translation (via IRES on promoter) of ATF-4 which translocates to the nucleus and induces the expression of genes involved in antioxidant responses, amino acid metabolism, autophagy, apoptosis as well as ER chaperones^28^,^29^,^30^. Assessing the ER stress pathway following NIR laser treatments, sub-phototoxic doses were noted to increase ATF-4, BIP, GADD34, cleaved ATF6 and phosphorylation of e-IF2α while phototoxic doses suppressed this pathway (Fig. 4a,c). Nuclear translocation of ATF-4 was observed at sub-phototoxic laser doses (Fig. 4b) while IRE1α and XBP1 did not appear to be modulated (Supplementary Fig. 5b,c).

We next examined the role of autophagy and HSPs following laser treatments. Treatment of sub-cytotoxic doses of laser leads to activation of autophagy and HSP70 in a dose dependent manner (Fig. 4c and Supplementary Figs. 6a–d). However, phototoxic laser dose appears to suppress ATF-4 and HSP70, but not HSP90, and induces excessive autophagy as evident by increased LC3 cleavage in both the cell lines (Fig. 4c and Supplementary Fig. 6a,b). Interestingly, HSP70 inhibition, and not HSP90, inhibition demonstrated increased sensitivity (lower dose) to laser phototoxicity (Fig. 4d). To further explore the roles of ER stress and autophagy in laser phototoxicity, cells were pre-treated with autophagy inhibitors (3MA and Bafilomycin A1) or low concentrations of ER stressors (Lipopolysaccharides, Phorbol myristate acetate, Tamoxifen citrate or Rapamycin). Pretreatment with autophagy inhibitors sensitized cells to lower doses of laser while priming cells with low concentrations of ER stressors had a cytoprotective effect (Fig. 4e,f and Supplementary Fig. 6e). Strikingly, cells pre-treated with high concentrations (low doses were protective) of ER stressor, Rapamycin was unable to protect cells from laser phototoxicity (Fig. 4g) that correlated with ATF-4, HSP70 and cleaved LC3 levels (Fig. 4h).

### ATF-4 is a master regulator of cellular stress response

**Figure 5:**
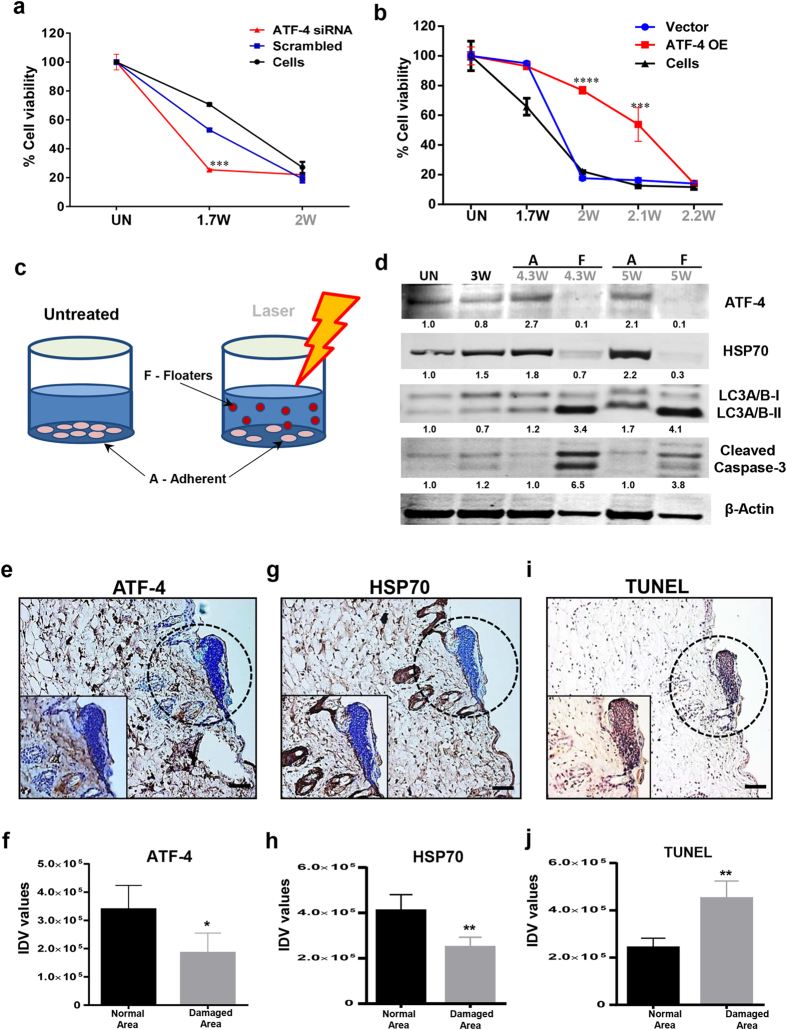
ATF-4 protects cells from cellular stress. HaCaT cells were transfected with ATF-4 siRNA and were treated with different laser doses. Cellular viability (**a**) (n = 3) was assessed at 24 hrs. (**b**) Conversely, over expressing ATF-4 stable HaCaT cells was treated with increasing doses of laser and cellular viability was assessed. Statistical significance was determined using two-way ANOVA among different treatments using the Bonferroni’s multiple comparison test (n = 3). (**c**) Outline shows HaCaT cells treated with phototoxic dose of laser that generates two populations, surviving cells that remain adherent (A) and dying or dead cells that float (F) in media. These were collected separately, lysed and immunoblotting (**d**) was performed to assess ER stress pathway. Histological assessment of mice skin showing damaged area in laser-treated skin was performed by immunohistochemistry for ATF-4 **(e**,**f**), HSP70 (**g**,**h**) and (**i**,**j**) TUNEL positivity. (Scale bar = 70 μm). Quantitation of high power images from mice sections are plotted (n = 5). Insets show high power magnifications. Images in a gel are cropped (horizontally) and placed together for better clarity of results and were run in the same experimental conditions. Significance was assessed with paired Student’s *t*-test. Statistical significance are indicated as *P* *<* 0.05 (*), <0.001 (**), <0.0001 (***) and <0.00001(****).

ATF-4 is known to play a critical role in ER stress responses^31^. We next examined the specific role of ATF-4 in mediating the cellular stress response in laser phototoxicity. ATF-4 was knocked down in both HaCaT and NOKSI cells with siRNA and 70-80% knockdown was achieved (Supplementary Fig. 7a). These cells were challenged with increasing laser doses and demonstrated increased sensitivity to laser phototoxicity (Fig. 5a and Supplementary Fig. 7b). Concurrently, laser treated ATF-4 knockdown cells showed reduced HSP70 levels, but had increased cleaved LC3 levels (Supplementary Fig. 7c). To further validate the role of ATF-4, overexpression studies were performed. As both HaCaT and NOKSI cells appear to ubiquitously maintain high levels of ATF-4 expression, a maximal 2-fold overexpression was achieved in multiple, viable, stable clones (Supplementary Fig. 7d,e). ATF-4 overexpression clones were observed to be resistant to laser phototoxicity, requiring higher doses to demonstrate cytotoxicity (Fig. 5b and Supplementary Fig. 7f). To corroborate the pro-survival role of ATF-4 in stress induced cells, two fractions of cells, either floaters (dead/dying) or adherent (surviving), were examined after 24 hours following phototoxic laser treatments (Fig. 5c). The floaters demonstrated low levels of ATF-4 and HSP70 along with increased cleaved LC3A/B and cleaved caspase-3 while the adherent cell fraction showed the opposite expression profile (Fig. 5d).

### *In vivo* validation of laser phototoxicity

**Figure 6:**
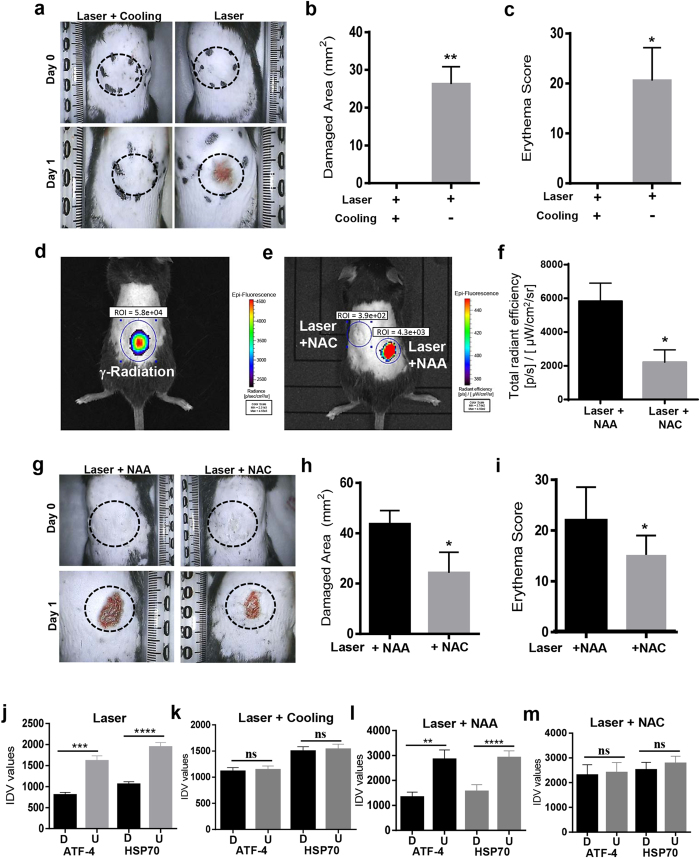
Laser mediated damage is via heat and ROS *in vivo*. (**a**) Images of the dorsal portion of 5-week-old C57BL/6NCr male mice treated with laser (55 °C) or following skin cooling for 30 s. Quantitation of damaged area (**b**) and erythema score (**c**) are shown (n = 5). (**d and e**) Representative image of Reactive Oxygen Species (ROS) induction as measured with ROSstar probe using IVIS *in vivo* imaging is shown (Radiation is used as a positive control). Treatment sites were pretreated with NAC or NAA and ROS level was quantitated after laser treatment (**f**). Dorsal area was photographed (**g**) and damaged area (**h**) and erythema (**i**) are shown (n = 5). (J-m) Laser treated mice skin tissues from these experiments were assessed with immunostaining for ATF-4 and HSP70 expression and their quantitation was performed with ImageJ (n = 5). Significance was assessed with paired Student’s *t*-test and denoted as *P* *<* 0.05 (*), <0.001 (**), <0.0001 (***), <0.00001(****) and not significant (n.s.).

To validate the observed molecular mechanism of laser phototoxicity *in vivo*, laser damaged mice skin were assessed for ATF-4 and HSP70 and demonstrated decreased expression compared to surrounding, undamaged areas (Fig. 5e–h and Supplementary Fig. 7g, 8a). The damaged tissues were noted to demonstrate increased TUNEL positivity (Fig. 5i,j). As the prior *in vitro* studies had observed that either neutralization of rise in temperature or ROS generation could rescue laser phototoxicity, we next examined these effects *in vivo*. Pre-treating the target tissues with NAC or pre-cooling was able to abrogate laser damage (Fig. 6a–i and Supplementary Fig. 8b). Expressions of ATF-4 and HSP70 in the heat and ROS neutralization skin specimens were retained correlating with decreased tissue damage observed (Fig. 6j–m and Supplementary Fig. 8c,d). Together, these results indicate that laser phototoxicity *in vitro* and *in vivo* involves heat and ROS-generated damage that results in ER stress mediated by ATF-4 levels.

## Discussion

Therapeutic applications of various wavelengths and higher doses of light are popular in current medicine. However, clinical applications of low doses of visible and near-infrared light have shown variable clinical efficacy. The primary photochemical event mediating PBM appear to involve generation of Reactive Oxygen Species (ROS) following absorption by various cellular chromophores, especially cytochrome C oxidase in the mitochondria^8^,^32^,^33^. The ROS thus generated has the ability to activate several extracellular and intracellular biological pathways^34^. A significant barrier in enabling more widespread use of PBM therapy is the lack of our understanding of target tissue parameters and biological responses that has prevented our ability to outline precise device (source, wavelength, dose and delivery) parameters for effective clinical treatment protocols. This study was motivated by the lack of a precise therapeutic dose limit, molecular pathway of phototoxicity and paucity of safety data for the use of NIR lasers. In elucidating the mechanisms of laser phototoxicity, we noted that surface temperature (45 °C) and treatment time (30 sec) correlated with significant skin damage irrespective of skin color and conventional laser treatment parameters namely, irradiance and fluence. Strikingly, *in vitro* laser treatments were similarly delivered with varying (distance, irradiance, time) parameters but also demonstrated a striking correlation with treatment temperature. This suggests that monitoring surface temperature could be a real time, *in vivo* clinical biomarker to monitor the detrimental (phototoxic) effect of NIR laser applications.

Previous studies have postulated that the levels of ROS generation could determine the transition from the therapeutic to detrimental biological responses^22^. A similar phenomenon has been observed by toxicologists using various doses of environmental agents termed Hormesis^35^,^36^. Indeed, our previous study noted one of the beneficial effects of low amounts of laser-generated excessive ROS involves activation of latent TGF-β1 and promotes wound healing and regeneration^34^. Laser generated ROS and concomitant rise in temperature appear to act together to generate phototoxicity. Neutralization of heat or ROS rescues phototoxicity both *in vitro* and *in vivo*. The increase in laser-induced tissue temperature appears to reduce activity of crucial cellular ROS neutralizing enzymes Catalase and Glutathione Reductase, which lead to detrimental oxidative damage as shown previously^37^,^38^,^39^. As excessive ROS could also damage DNA generating genotoxicity^40^,^41^, this potentially raises significant clinical concerns for the use of both high power (with low doses in peri-treatment zones) and low power NIR laser applications. Careful analyses of NIR laser treatments at both phototoxic and sub-phototoxic treatment zones using a wide range of cell and molecular analyses did not demonstrate any evidence for genotoxicity or mutagenicity.

However, ROS and thermal damage did result in an endoplasmic reticulum (ER) stress response. ER performs the dual role of protein folding and maturation as well as monitoring for cellular stresses due to misfolded or damaged proteins^42^. We observed that increasing laser doses induces ER stress and autophagy mediated via ATF-4 and HSP70. At the phototoxic dose threshold, we noted that ATF-4 expression was reduced along with HSP70 and concurrent increase in autophagy and apoptosis. Strikingly, pre-induction of mild ER stressors appear to prime the autophagy and cell repair response contributing to increased laser phototoxic dose threshold, a phenomenon termed Preconditioning^43^,^44^. Similar results were obtained with ATF-4 knockdown (sensitizes) and overexpressing cells (resistant) highlighting its role in protecting epithelial cells from laser induced stress. Studies with the ATF-4 knockout mice has shown conflicting results with both pro-survival^28^,^45^ and pro-apoptotic^46^,^47^ roles in distinct pathophysiological contexts. These knockout mice are born with multiple developmental defects, have low fertility and do not survive beyond a few weeks after birth^48^,^49^,^50^. Future studies to assess the *in vivo* roles of ATF-4 mediated laser phototoxicity necessitate generation of inducible, tissue specific mice models that are currently unavailable.

Given the subtle clinical response with current PBM treatments, the ability to neutralize laser phototoxicity by cooling target site or preconditioning with a low amount of stressors, raises interesting avenues to increase therapeutic dosing. Similar approaches are being actively explored in dermatological applications (pigmented lesions and hair removal) and radiation oncology^51^,^52^,^53^,^54^. In summary, this study provides fundamental insights into near-infrared light-biological tissue interactions and specifically the molecular phototoxicity pathway. Further, the clinical and molecular NIR phototoxicity biomarkers outlined in this study could facilitate development of safe and effective use of low power, near-infrared lasers for various clinical applications.

## Methods

### Cell lines

Human dermal keratinocytes (HaCaT) cells and human normal oral keratinocyte (NOKSI) cells were maintained in DMEM (Sigma-Aldrich, USA) supplemented with 10% fetal bovine serum (Invitrogen corporation, USA) along with 100 units/ml penicillin and 100 μg/ml streptomycin (Invitrogen Life Sciences, USA). Cells were grown at 37 °C in a humidified chamber with 5% CO_2_.

### Animal studies

All experiments were performed in accordance with the institute guidelines and approved by the animal care and use committee (ASP#13-693). The dorsal skin of 5 weeks old C57BL/6NCr male mice (NCI Frederick) were shaved and naired. A baseline erythema and melanin score was obtained using a Derma lab probe (Cortex Technology, USA). An infrared camera ICI7640 (Infrared Cameras Incorporation, USA) was used to measure surface temperature of skin. Laser (810 nm diode) (AMD Lasers, USA) treatment was performed on the left and right dorsal skin of the mouse, ensuring the spine was avoided. The laser probe was setup 2 cm perpendicular to the mouse with a spot size of 2 cm in diameter (Irradiance = 1 W/cm^2^ and fluence = 21 J/cm^2^). The laser was used for various treatment time based on the melanin score and dynamically adjusted (laser switched on/off) to maintain specific surface temperature (45-55 °C) as monitored by the IR camera. For higher temperature (>55 ^o^C), the probe was moved continuously in a controlled manner to prevent excessive heating as shown in Supporting Video 1. For neutralization experiments, laser treated area was either cooled with cryogen spray cooling (1,1 Difluoroethane Falcon Safety Products, Inc., USA) or N-Acetyl Cysteine (NAC, 100 mM) or N-Acetyl Alanine (NAA, 100 mM) (Sigma-Aldrich, USA) applied topically as a gel (OptixCare, Eyelube, USA) or injected subcutaneously (100 μl) 15 min prior to laser treatments.

### Laser treatments in cell culture

Laser treatments were performed using an 810 nm, continuous wave GaAlAs laser (AMD lasers, USA). Treatments were given at a distance of 10 cm or 14.5 cm for 96 well and 6 well plate, respectively, such that the spot size covered treatment surfaces. To assess phototoxicity, the bottom surfaces of 96 well (black well clear bottom) or 6 well tissue culture plate were covered with a black rubber mastic tape (Scotch, USA). Cells were treated in 96 well and 6 well plates containing 200 μl and 2 ml media, respectively. Various irradiances (W/cm^2^) were used to generate phototoxicity by adjusting power and distances as outlined in Supplementary Table 4. Following laser treatments, cells were assessed for viability with AlamarBlue dye (Life Technologies, USA) at 24 hours. Laser doses that result in ≥70% cytotoxicity of cells in the laser treatment were considered as phototoxic dose. For neutralization experiment cells were pre incubated with NAC (1 mM), Catalase (1000units/ml) (Sigma-Aldrich, USA) for 2 hrs, followed by laser treatment. For the helium treatments, helium gas (Worthington, USA) was bubbled through media for 5 min and decreased oxygen percentage (311 mV to 200 mV) was verified by a redox probe (Redox/ORP electrode, Orion, Thermo Scientific, USA).

### Surface temperature measurements

Surface temperature during the laser treatments were measured using IR camera (*in vitro* and *in vivo*). Measurements using IR camera (ICI7640, Infrared Cameras Incorporation, USA) was used to non-invasively measure surface temperature of skin or cell culture plates in real time (one frame per second) using IR flash software (version 2.14.19.5 Infrared Cameras Incorporation, USA) with accuracy of ±1 ^o^C.

### Cell viability assay

Cellular viability after laser treatment was measured using AlamarBlue dye (Thermo scientific, USA)). The AlamarBlue dye is an oxidized form of resazurin dye that is blue in color and non-fluorescent but if incubated with viable cells, the reagent changes color from blue to red and becomes fluorescent which can be measured at 530/590 (excitation/emission)^55^. Percent viability was calculated using the following formula: % Viability = (Fluorescence of treated cells/Fluorescence of untreated cells) x 100.

### Tunel assay

Apoptosis, characterized by the genomic DNA fragmentation that can be detected by labeling the terminal end of nucleic acids using Terminal deoxynucleotidyl transferase dUTP Nick End Labeling (TUNEL), was detected with a kit (TACS 2TdT-DAB *In situ* Apoptosis Detection kit, Trevigen, Inc., USA)^56^. For adherent cells, cells cultured in glass chamber slides (Lab-TekII, Nunc, USA) were treated and fixed in 3.7% buffered formaldehyde (Sigma-Aldrich, USA) followed by the labeling reaction. For tissues, sections were deparaffinized in xylene (Sigma-Aldrich, USA) for 15 minutes and then transferred to absolute alcohol for 10 minutes followed by incubation in Phosphate buffered saline (1x PBS) (Life Technologies, USA) for 10 minutes. Tissue or adherent cells samples were then incubated with 50 μl of Proteinase K solution at 37 °C for 30 minutes followed by two washes in deionized water. Endogenous peroxidase activity was then blocked using 5% hydrogen peroxide in methanol and washed in PBS for 1 minute. Samples were incubated in TdT labeling buffer for 5 minutes and then incubated with 50 μl of labeling reaction (containing TdT dNTP, TdT Enzyme, 1X Manganese Cation and TdT labeling buffer) for 1 hr at 37 °C. Reaction was stopped using TdT stop buffer for 5 minutes. Samples were washed twice in deionized water for 5 minutes each. Further, tissue samples were incubated with 50 μl of Strep-HRP solution (secondary) for 10 minutes at 37 °C followed by two washes in PBS for 2 minutes each. Finally, the colorimetric substrate diaminobenzidine (DAB) and enhancer H_2_O_2_ were used followed by counterstaining with Haematoxylin and mounted using Toluene-based mounting media (TBS, SHUR/Mount, USA).

### ROS detection

#### In vitro assay

Quantitation of Reactive Oxygen Species (ROS) generated by laser treatments was performed by DCFDA staining and flow cytometric analysis^57^. The CM-H_2_DCFDA (Molecular Probes, USA) is a membrane permeable molecule which passes easily through the cell membrane and increase in its fluorescence signal can be observed upon stimulation by an ROS inducing agent. HaCaT or NOKSI cells were trypsinized and 1 × 10^6^ cells per ml suspension was made in PBS. 200 μl of the above cell suspension were treated with phototoxic dose in clear and dark tube (2 W at 10 cm). The cells were then incubated with 1 μM DCFDA solution in DMSO in dark for 5-10 minutes at room temperature. The distribution of DCFDA stained HaCaT and NOKSI cells were determined by flow cytometry (BD, FACS Canto, USA) using the FL-1 channel (Excitation/Emission: 492–495 nm/517–527 nm).

#### In vivo assay

Detection of ROS in mice was performed using an *in vivo* fluorescent ROS probe (ROSstar 800CW probe, Licor, USA). ROSstar is a hydrocyanine (reduced dyes) based probe design to detect extracellular reactive oxygen species. Immediately after laser treatment 50 μl of ROSstar probe was injected subcutaneously and incubated for 20 min. After the incubation fluorescence was detected using the IVIS (Caliper, USA) *in vivo* imaging system.

#### Glutathione Reductase (GR) assay

Glutathione Reductase activity was measured by the increase in absorbance due to reduction of DTNB [5,5’-dithiobis(2-nitrobenzoic acid)] at 412 nm (Colorimetric assay), as per the manufacturer protocol (GRSA, Sigma-Aldrich, USA). Briefly, 0.6 million per 20 μl of HaCaT and NOKSI cells were exposed with phototoxic dose in clear and black well plates. Cells were lysed (5-10 min after exposure) using 0.1% Triton X-100 and color development was performed using reaction mixture containing oxidized glutathione (1 mM), DTNB (0.7 mM), NADPH (40 μM) in assay buffer. The reaction was started by the addition of NADPH solution to the remaining mixture. GR activity was calculated by the following formula: Units/ml = (Change in Abs. sample – Change in Abs. blank) x (dilution factor)/ε^mM^ x (volume of sample in ml); For NADPH *ε*^mM^ = 6.22 mM^−1^cm^−1^; For TNB6 *ε*^mM^ = 14.15 mM^−1^cm^−1^.

Activity of purified Glutathione Reductase enzyme was assessed in clear and black well plates following phototoxic laser treatments. Glutathione Reductase pure enzyme (EC 1.6.4.2) in potassium phosphate buffer, pH 7.5, with EDTA and trehalose as a stabilizer (G0665, Sigma-Aldrich, USA) was used for the assay. This GR solution containing >1 unit per ml (20 μl) was directly exposed to laser treatment in clear and black well plates and enzymatic activity was assessed.

#### Catalase Assay

Catalase enzyme converts hydrogen peroxide to water and oxygen (catalytic pathway) and hence this assay is based on the measurement of hydrogen peroxide substrate remaining after the action of catalase on the cellular lysate. This colorimetric method uses a substituted phenol (3,5-dichloro-2-hydroxybenzenesulfonic acid), which couples oxidatively to 4-aminoantipyrine in the presence of hydrogen peroxide and horseradish peroxidase (HRP) to give a red quinoneimine dye (N-(4-antipyryl)-3-chloro-5-sulfonatep-benzoquinone-monoimine) that absorbs at 520 nm (CAT100, Sigma-Aldrich, USA). Absorbance of the red quinoneimine dye versus amount of H_2_O_2_ standard curve provides catalase activity in μmoles/min/ml by using the following formula: Activity (μmoles/min/ml) = [Change in μmoles (H_2_O_2_) x dilution of sample x 100]/[V (Sample volume in ml) x reaction time]. For assessing the effect of phototoxic dose on pure enzyme, Catalase enzyme (C8362, Sigma-Aldrich, USA) was diluted (dilution 1:10,000) in enzyme dilution buffer and treated with 810 nm laser at phototoxic doses followed by the color development as described above.

### Statistical Analyses

Statistical analyses were performed in Graphpad prism software (GraphPad Software, Inc., USA). Significance among two groups was assessed using paired Student’s t-test while multiple groups were assessed using analysis of variance (ANOVA) with Bonferroni’s Multiple Comparison Test. All treatments were compared to untreated control and *p* < 0.05 was considered significant. *P* value are indicated in figures as <0.01 (*), <0.001 (**), <0.0001 (***), <0.0001 and not significant (n.s.) respectively.

## Additional Information

**How to cite this article**: Khan, I. *et al.* Molecular pathway of near-infrared laser phototoxicity involves ATF-4 orchestrated ER stress. *Sci. Rep.* **5**, 10581; doi: 10.1038/srep10581 (2015).

## Supplementary Material

Supplementary Information

Supplementary Video 1
