# Supplementary material for: Molecular pathway of near-infrared laser phototoxicity involves ATF-4 orchestrated ER stress
Source: Sci Rep. 2015 Jun 1;5:10581. doi: 10.1038/srep10581 (PMC4450753; doi:10.1038/srep10581)
Supplement: Supplementary Information [file srep10581-s1.pdf]

SUPPORTING FILES

Molecular pathway of near-infrared laser phototoxicity involves ATF-4 orchestrated ER stress

Imran Khan, Elieza Tang and Praveen Arany

Cell Regulation and Control Unit, National Institute of Dental and Craniofacial Research, National Institutes of Health, Bethesda MD

Contents

Supplementary Figures

Supplementary Tables

Supporting Methods

References

Supplementary Figure 1

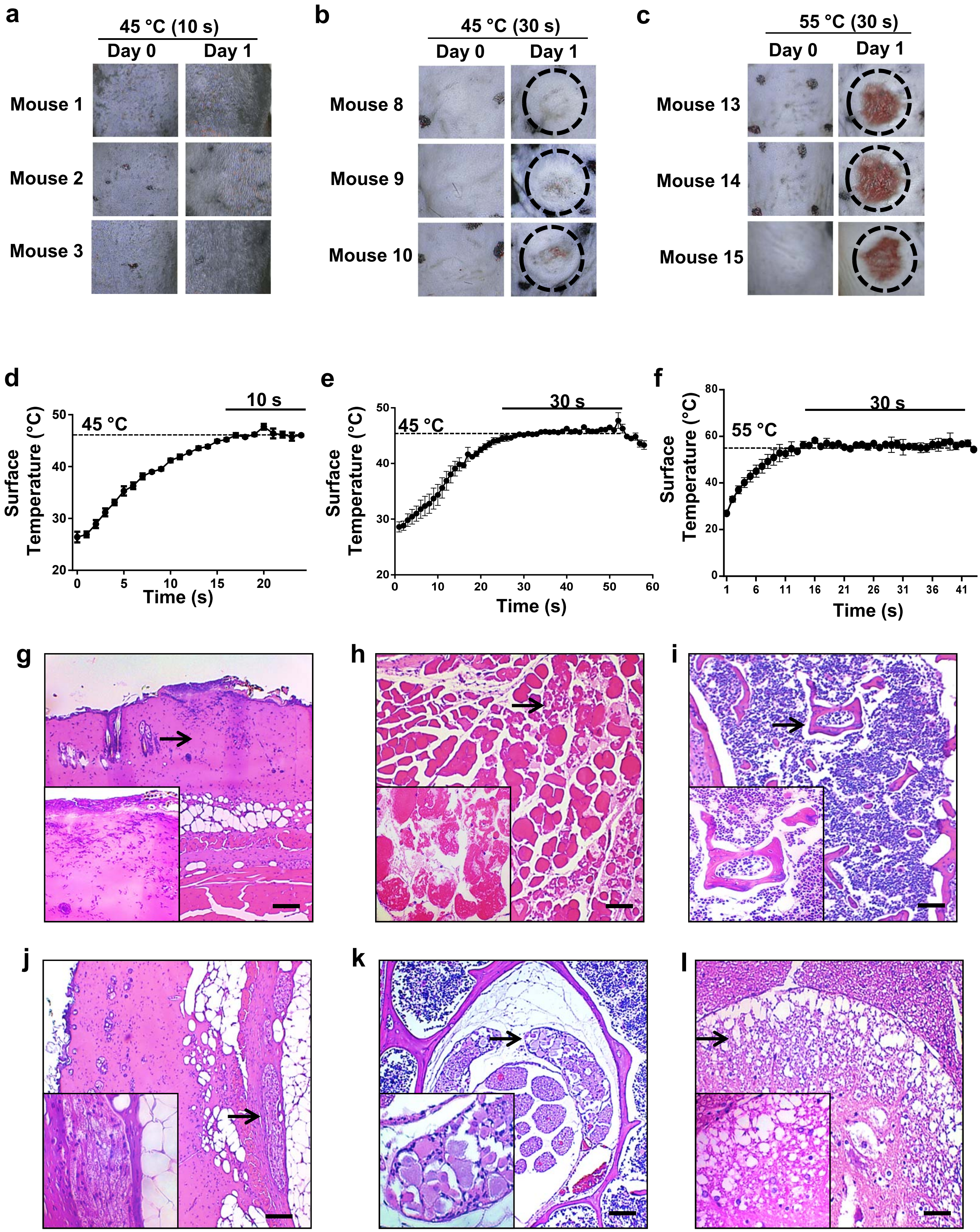

Supplementary Figure 2

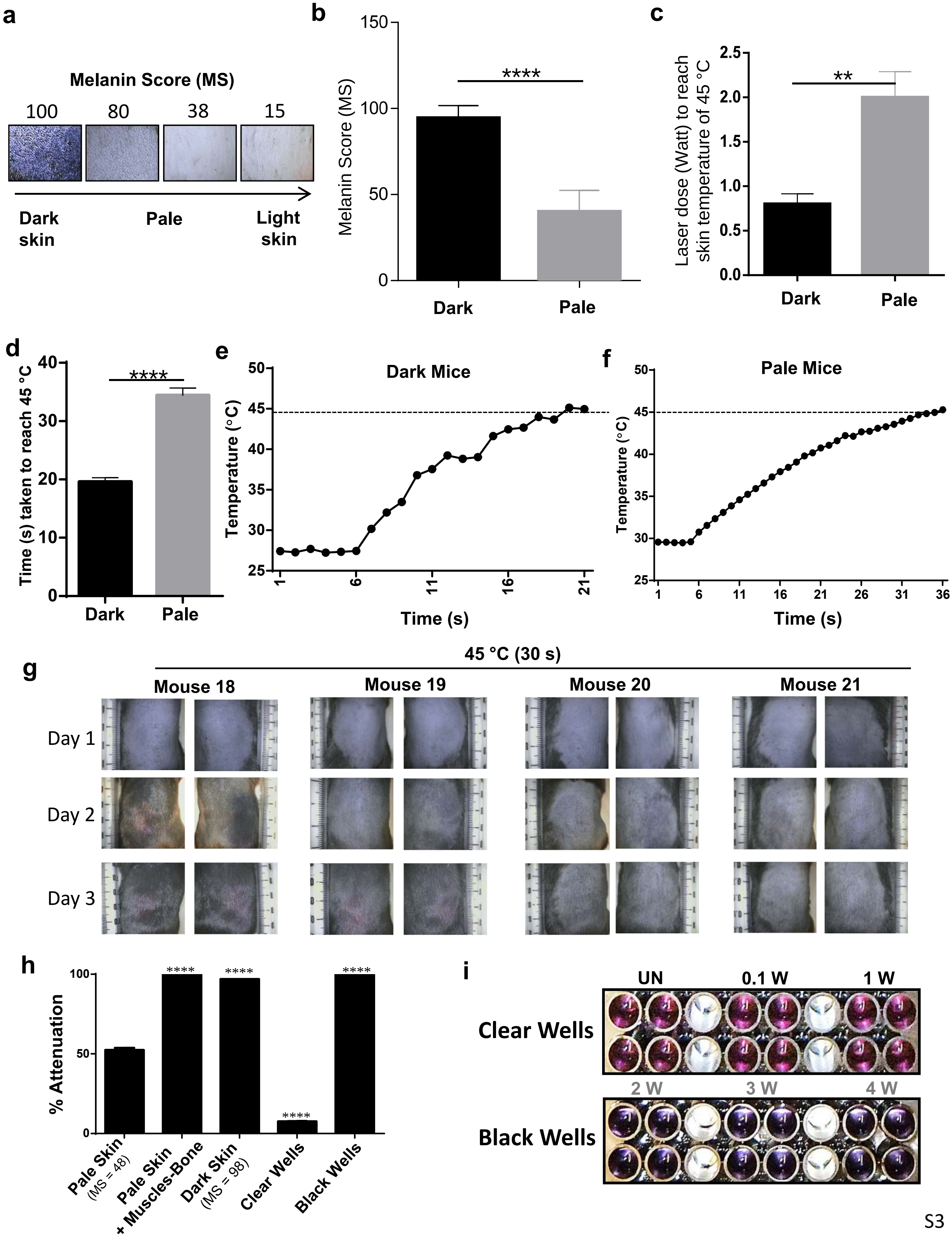

Supplementary Figure 3

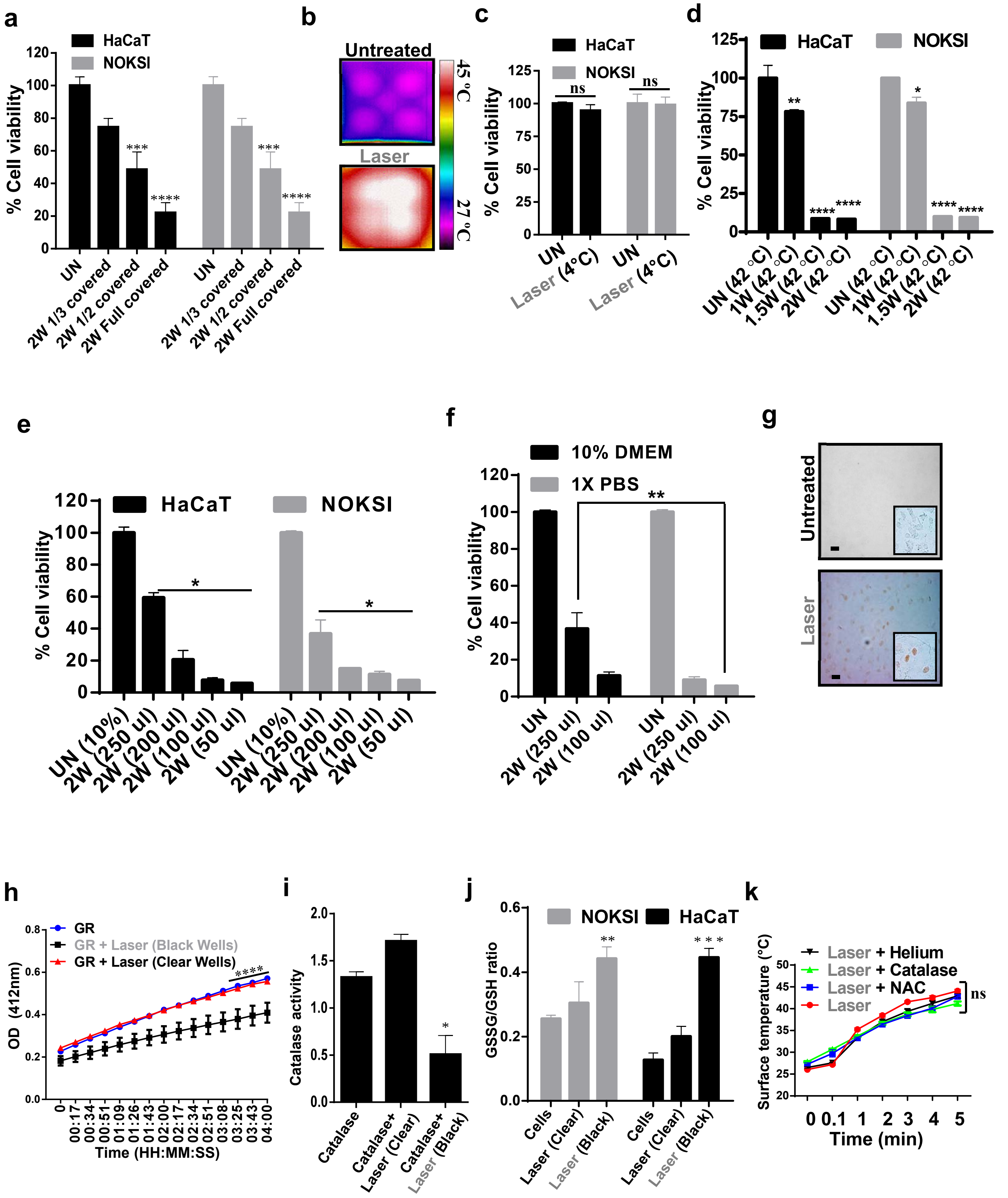

Supplementary Figure 4

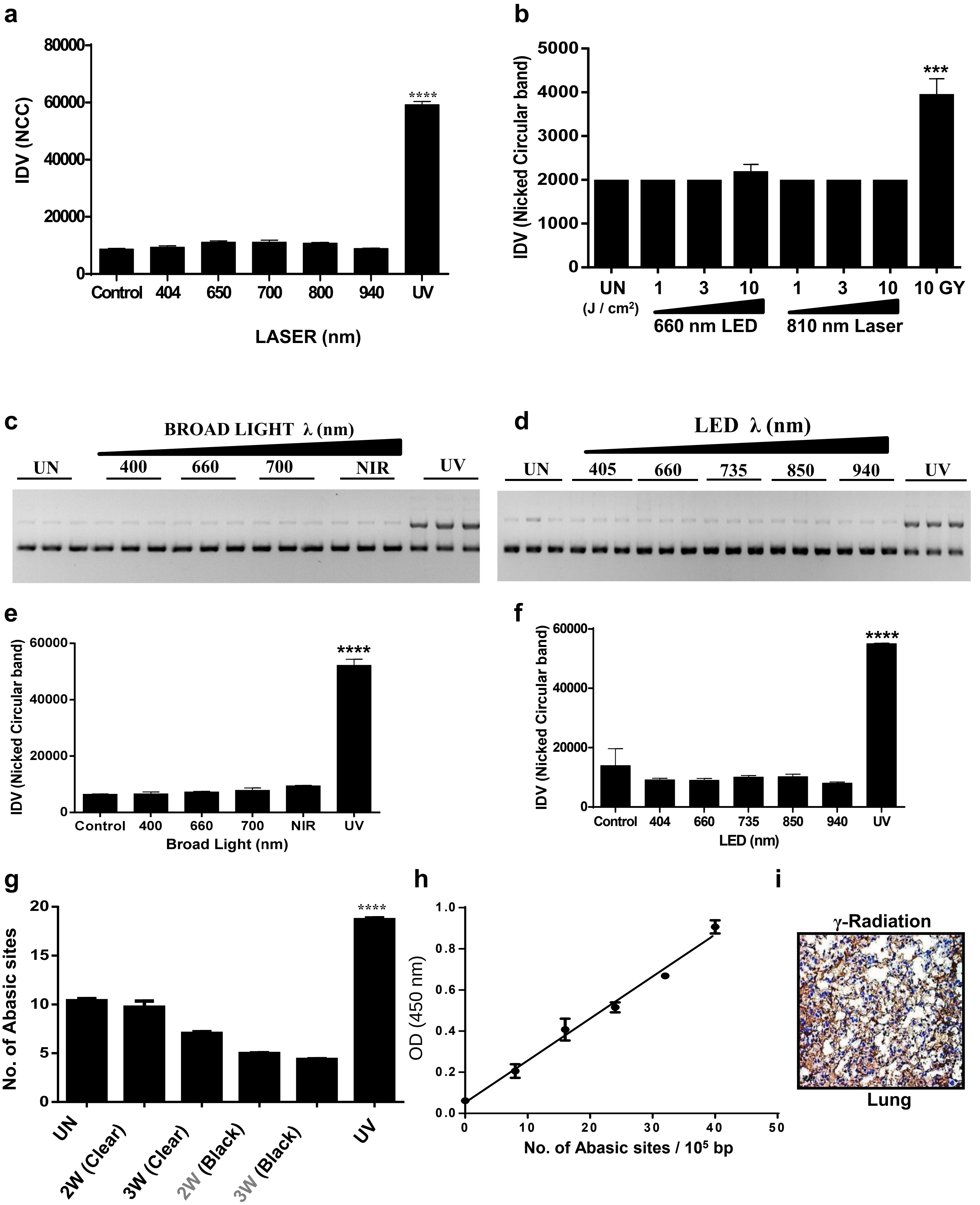

Supplementary Figure 5

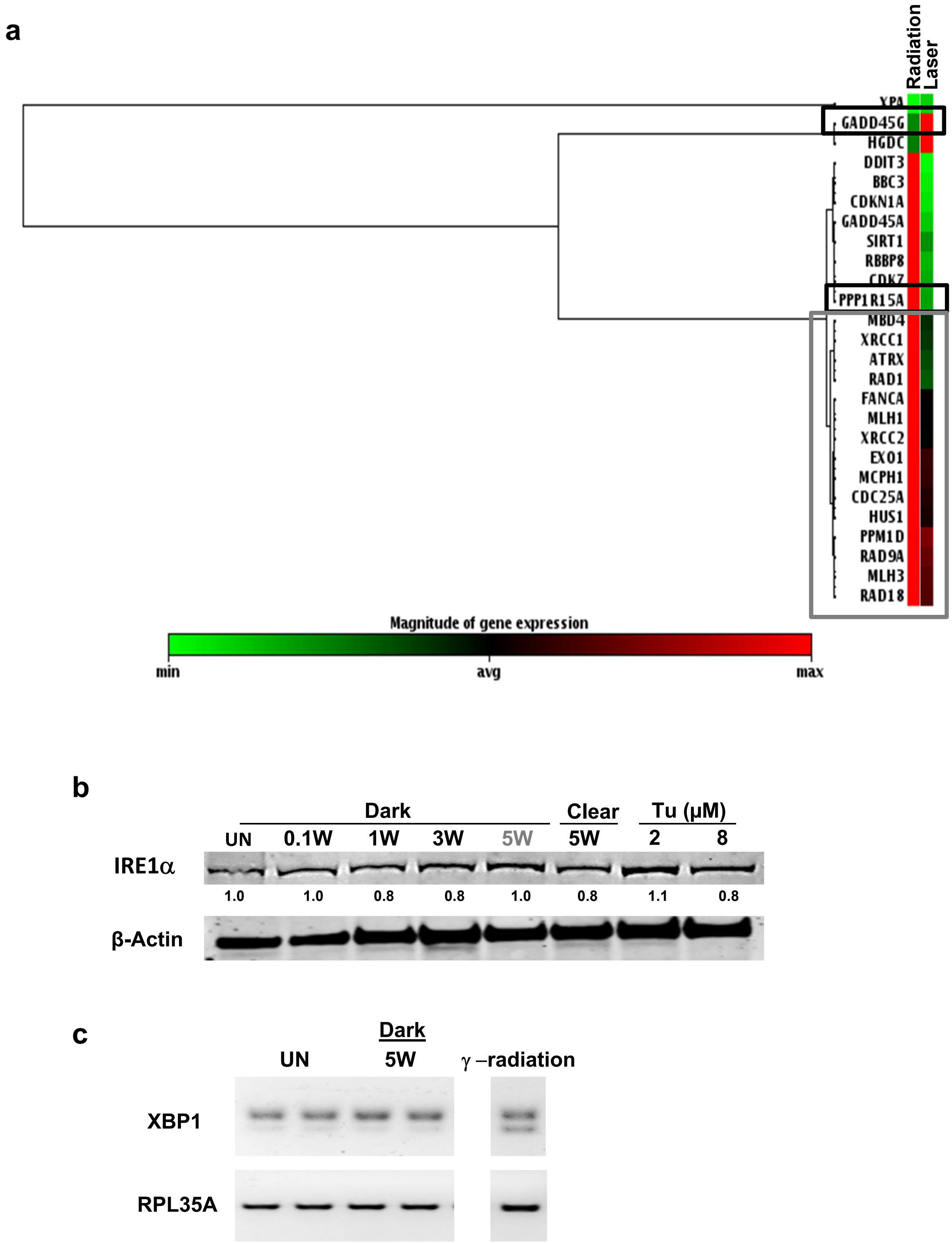

Supplementary Figure 6

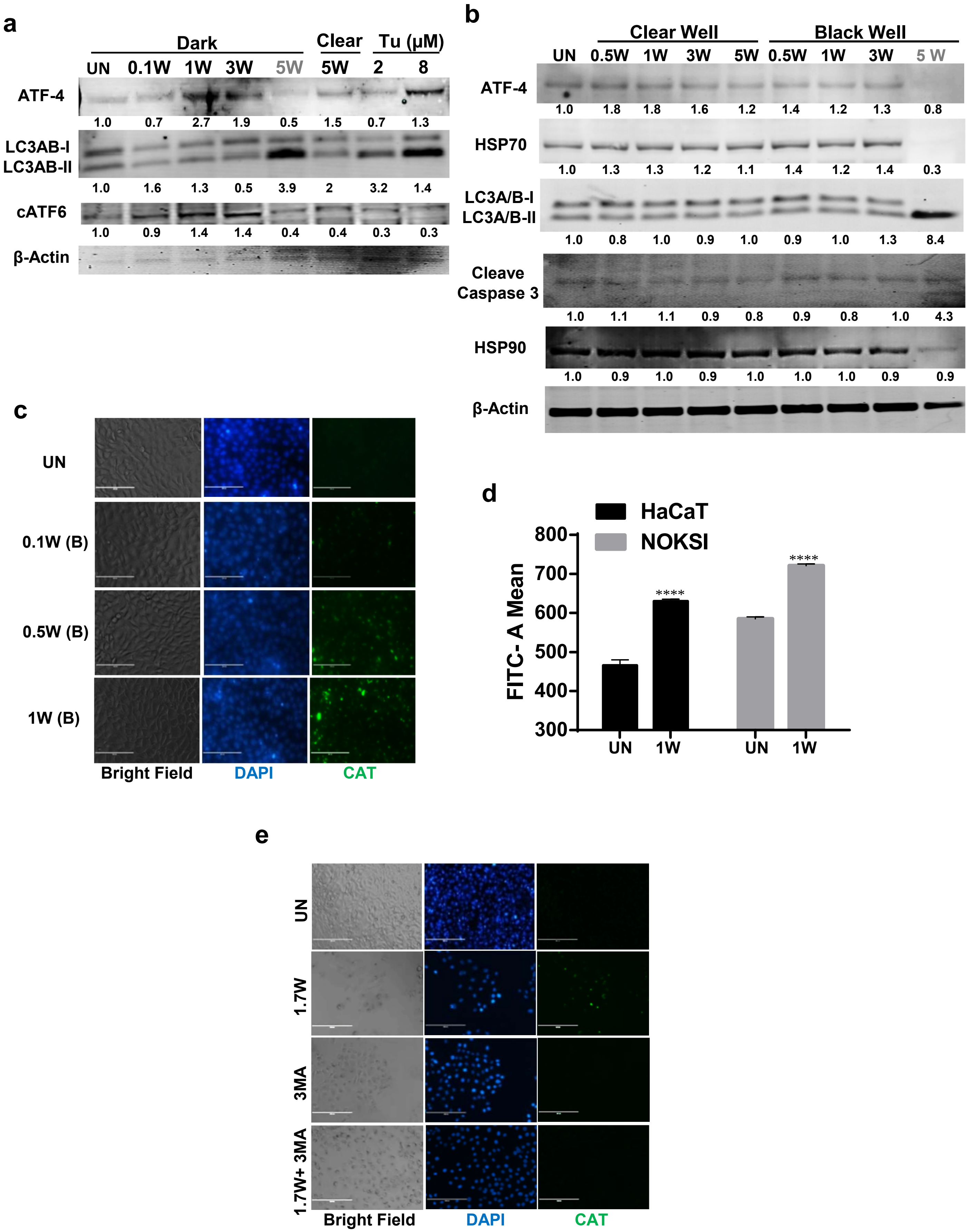

Supplementary Figure 7

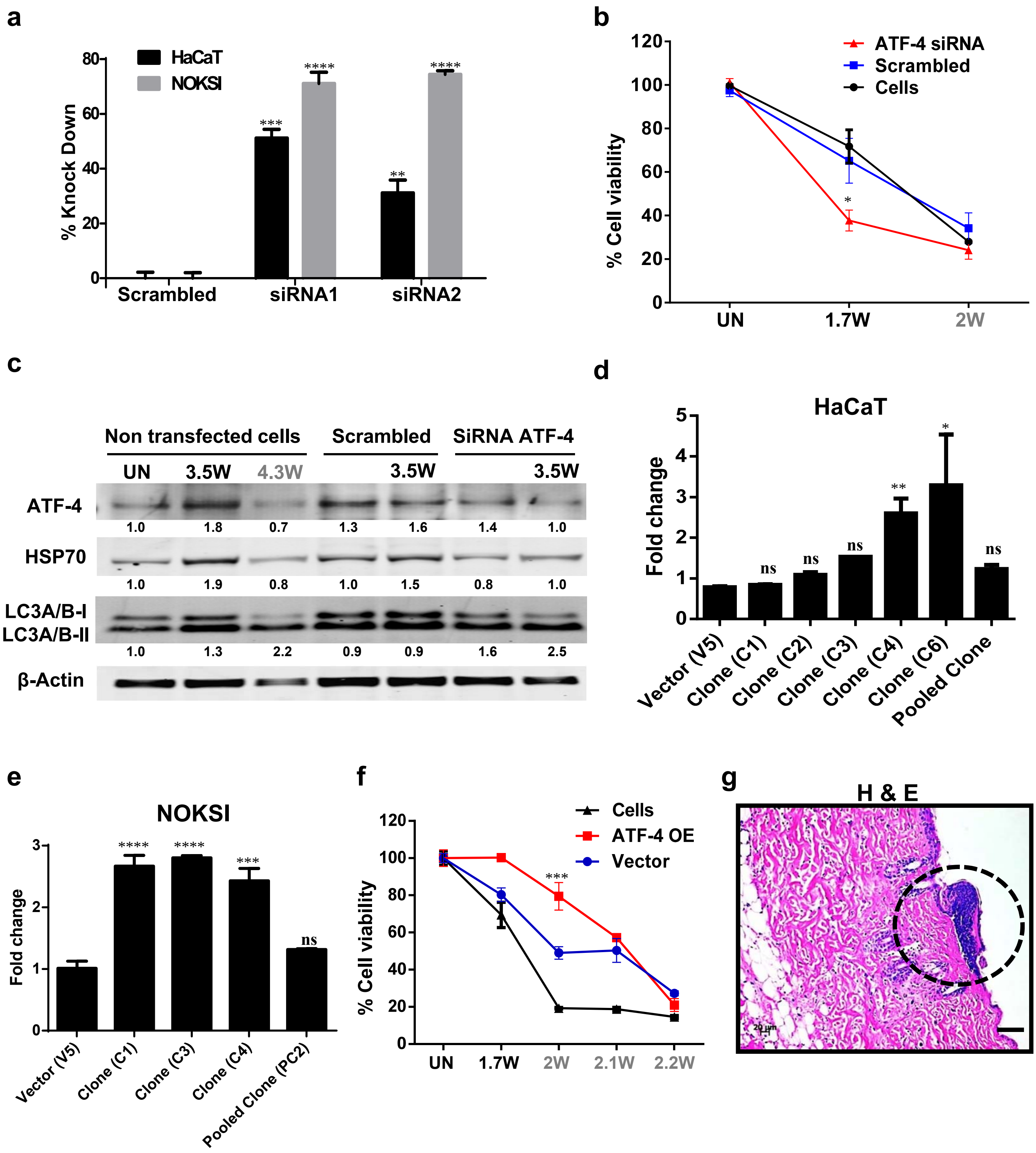

Supplementary Figure 8

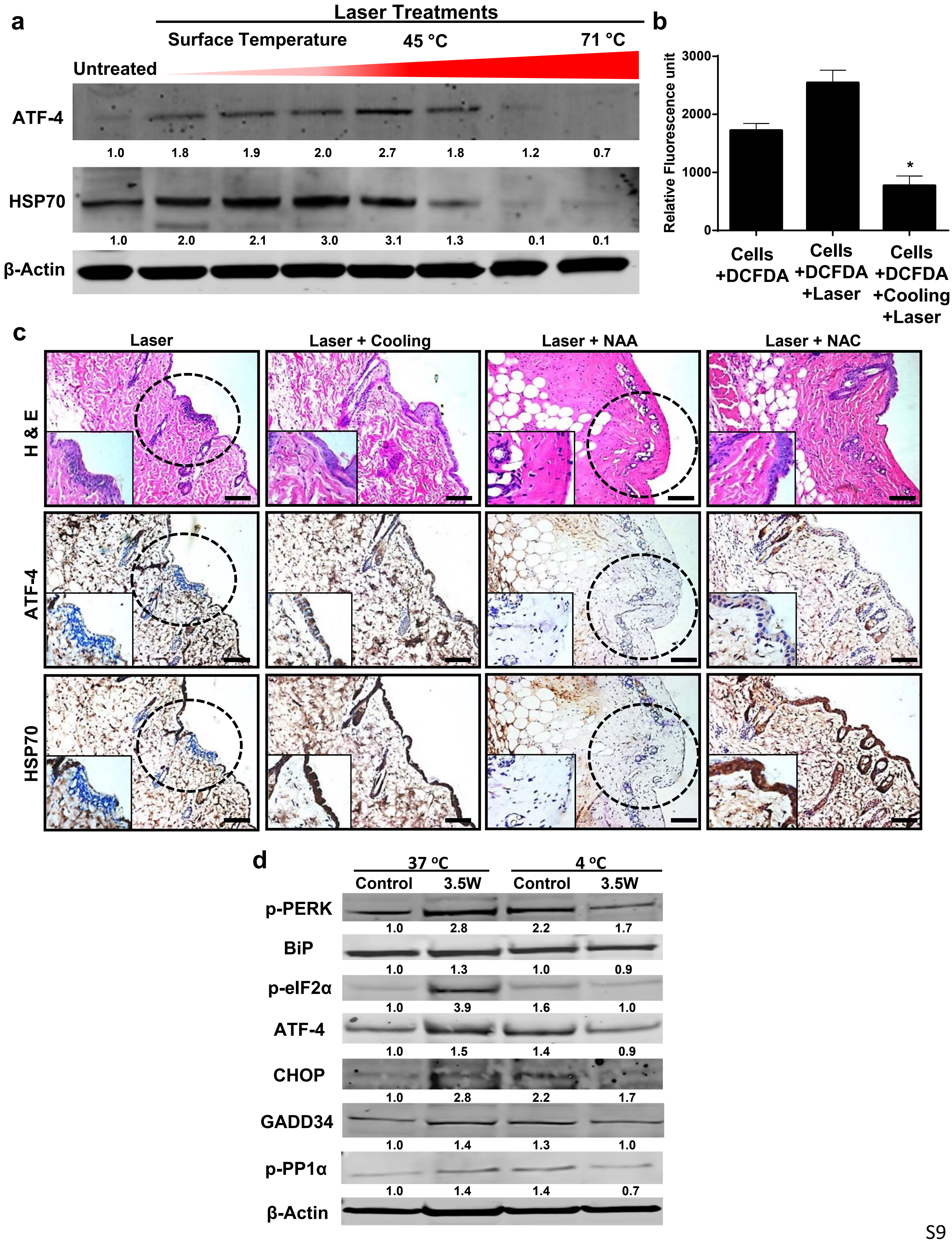

Supplementary Table 1

**Surface Temperature**	**< 45 °C**	**45 °C**	**45 - 55 °C**	**> 55 °C**
Immediately Post-treatment	----	----	Mild Edema (pallor)<br>Erythema<br>Damaged area	Prominent Edema<br>Severe Erythema<br>Damage area (burn)<br>*Leg Rise **
24 hrs Post-treatment	----	Mild Edema<br>Erythema<br>Damaged area	Prominent Edema<br>Prominent damaged area (Ulcer / wound)	Severe damaged area<br>Contractures / Paralysis<br>Death

* Leg rise is shown in Supplementary Video 1

Supplementary Table 1: Mice skin damage is temperature dependent

Dorsal skin of 5 week old C57BL/6NCr male mice were subjected to laser treatments at different temperatures and the type of damage noted immediate or post 24 hrs of treatment are outlined.

Supplementary Table 2

Position	Gene Symbol
A06	ATRX
A09	BBC3
B01	CDC25A
B03	CDK7
B04	CDKN1A
B12	DDIT3
C03	EXO1
C04	FANCA
C08	GADD45A
C09	GADD45G
C11	HUS1
D02	MBD4
D03	MCPH1
D05	MLH1
D06	MLH3
E07	PPM1D
E08	PPP1R15A
E10	RAD1
E12	RAD18
F05	RAD9A
F06	RBBP8
F11	SIRT1
G09	XRCC1
G10	XRCC2
H06	HGDC
G07	XPA

Supplementary Table 2: List of differentially induced genes by γ-radiation treatment

HaCaT cells were treated with radiation (10 Gy) and differentially induced genes (Fold change ≥ 2, n=2) after 6 hrs of treatment are presented, red font indicates upregulated genes while green font denotes down-regulated genes.

Supplementary Table 3

Position	Gene Symbol
C09	GADD45G
D06	MLH3
E07	PPM1D
E08	PPP1R15A
G05	TP73
H06	HGDC
G07	XPA

Supplementary Table 3: List of differentially induced genes by Laser treatment

HaCaT cells were treated with phototoxic dose of laser and representative differentially induced genes (Fold change $\geq$ 2) after 6 hrs of treatment are presented in the table. Red font indicates upregulated genes while green font denotes down-regulated genes.

Supplementary Table 4

6 well plate		96 well plate		Laser Irradiance (W/cm^2)	Fluence (J/cm^2)	Biological response
**Laser Power** (W)	**Distance** (cm)	**Laser Power** (W)	**Distance** (cm)			
3.2	14.5	1.5	10	0.069	20.7	***Sub-phototoxic*** (< 45 ^{O}C)
3.5	14.5	1.7	10	0.07	21	
4.3	14.5	2	10	0.090	27	***Phototoxic*** ($\geq$ 45 ^{O}C)
4.5	14.5	2.1	10	0.094	28.2	

Supplementary Table 4: Laser phototoxic and sub phototoxic doses in different plates

Laser irradiance (W/cm^2) at 10 and 14.5 cm was calculated using spot size diameter of 3.8 and 5.5 cm, respectively at 300 s to calculate fluence (J/cm^2). Increasing distance from 10 cm to 14.5 cm increased spot size area for treatment but reduces power meter detector surface coverage (20% to 10%) and, assuming Gaussian distribution, these were included in calculating final irradiance for dose studies.

SUPPORTING METHODS

GSH / GSSG ratio detection assay

GSH / GSSG ratio detection assay was performed using green fluorometric detection kit (Abcam, ab138881) which uses a proprietary non-fluorescent dye that becomes strongly fluorescent upon reacting with GSH (Ex / Em = 490 / 520 nm). An increased ratio of GSSG to GSH is an indicator of oxidative stress. For the detection of GSH/GSSG ratio, GSH and Total GSH assay was performed and subtraction of GSH from total GSH gives GSSG (oxidized form). This assay uses 50 µl of GSH Assay Mixture (GAM) into the wells containing GSH standard, blank control or test samples (0.6 million HaCaT and NOKSI cells) followed by incubation at room temperature for 30 min. Fluorescence was measured using a plate reader (Molecular Probes, USA).

Plasmid cleavage assay

Plasmid cleavage assay was performed using pUC19 plasmid (SD0061, 2686 bp, Thermo Scientific). Plasmid DNA (200 ng per reaction) was dissolved in 20 µl of TE buffer (10 mM Tris, bring to pH 8.0 with HCl and 1 mM EDTA) and treated with various doses of laser, LED and broad light. As positive controls, plasmid was exposed to UV rays for 10 min (UV stratalinker 1800, Stratagene, USA). Following treatments, plasmid DNA was analyzed by gel electrophoresis on 1% agarose gel[1] and images were captured using FluorChem™ E System (Cell Biosciences, USA).

DNA Damage (Apurinic site) Assay

The DNA damage assay (ab65353, Abcam) utilizes the ARP (biotinylated Aldehyde Reactive Probe) which reacts specifically with an aldehyde group present in the open ring form of the AP sites. Tagged AP sites are quantitated using avidin-biotin assay by colorimetric detection. HaCaT and NOKSI cells were treated with different doses of laser and genomic DNA was isolated (Qiagen, USA). Isolated genomic DNA (0.1 µg/µl) was mixed with ARP solution (5 µl each) and were incubate for 1 hr at 37 °C. Next, DNA was precipitated by adding 88 µl TE, 2 µl glycogen along

with 0.3 ml of pure ethanol and incubated at -20 °C for 10 min. Precipitated DNA was centrifuged and washed with 70% ethanol. DNA pellet was air dried for 5 min. The biotin-tagged DNA samples were dissolved in 1 ml of TE buffer (0.5 μg/ml). After sample preparation standards were also prepared by diluting the 40 ARP-DNA Standard (40 ARP sites per 10^5 bp). These solutions were mixed with 100 μl of the DNA binding solution and incubated over night at room temperature. Wells were washed with wash buffer and 100 μl diluted HRP-streptavidin solution was added to each well and incubated for 1 hr at room temperature. Following washes, HRP developer (100 μl per well) was added and incubated for 1 hr at room temperature. Color development was quantitated at OD 650 nm in a plate reader (Molecular Probes, USA).

Ames test

Ames test was performed in Muta-Chromo Plate kit (EBPI, 96 well microplate version) using TA100 (Base-Pair Substitutions) and TA98 (Frame Shift Mutations) strains of the *Salmonella typhimurium.* Ames test uses mutant strains of *Salmonella typhimurium* (TA100 and TA98), carrying mutation(s) in the operon coding for histidine biosynthesis. When these strains of bacteria are exposed to mutagenic agents, reverse mutation from amino acid (histidine) auxotrophy to prototrophy occurs, which could be detected by change in purple wells to yellow. Both the strains were incubated overnight at 37 ºC for 16 to 18 hours. An aliquot of 200 μl of both the strains were exposed with different dose of laser in black well plate in sterile hood. Following this, reagents A through E (A: Davis-Mingioli salts, B: D-glucose, C: Bromocresol Purple, D: D-Biotin, E: L-Histidine) were mixed [21.62ml (A) + 4.75ml (B) + 2.38ml (C) +1.19ml (D) + 0.06ml (E) for a total of 30.00 ml] and 2.5 ml of reaction mixture was dispensed into each tube. This reaction mixture was inoculated with treated or untreated bacterium, mixed well and 200 μl was dispensed into the each well of 96 well plate. For positive control 100 μl of sodium azide or 2-Nitrofluorene were added to reaction mix for TA100 and TA98 strains, respectively. Plates were incubated at 37 ºC for 5 days,

visually scored for number of revertants and significance was determined as per manufacturer's instructions.

RNA extraction, real-time RT-PCR and PCR arrays

HaCaT and NOKSI cells were lysed in RLT buffer (containing βME) and total RNA was extracted using Qiagen RNA isolation kit (Qiagen, USA) according to the manufacturer's protocol. For cDNA synthesis, 2 µg of RNA was reverse transcribed using ABI cDNA synthesis kit (Applied Biosystems, USA) and 1/100th of the reaction product was used per 20 µL PCR reactions. Real time PCR quantitations of ATF-4 (F- 5' AGTCCCTCCAACAACAGCAA 3' and R- 5' GAAGGTCATCTGGCATGGTT 3'; Tm 59.5 °C, 133 bp) and RPL35A (F- 5' AAGGGAGCACACAGCTCTTC 3' and R- 5' CTGGTTTTGTTTGGTTTGCC 3'; Tm 59.5 °C, 141 bp) was performed in ABI StepOne Plus sequence detection system (Applied Biosystems, USA). For semi-quantitative PCR of XBP1 (F-5' TTACGAGAGAAAACTCATGGCC 3' and R- 5' GGGTCCAAGTTGTCCAGAATGC 3'; 59.5 °C, 257/283 bp) ABI 9700 PCR machine was used. The reactions were performed using Dynamo^{TM} SYBERgreen mix (Finnzymes, Finland) in triplicate reactions. The differential expression was determined by the formula: δCT = CT gene-CT RPL; δδCT= δCT treated-δCT untreated; Fold Change= $2^{-\delta\delta CT}$. PCR array was performed using RT² Profiler™ PCR Array Human DNA Damage Signaling Pathway (Cat. No. PAHS-029ZC, Qiagen, USA). Reactions were performed using 20 ng of cDNA along with RT² SYBR Green ROX qPCR Mastermix (Cat. No. 330520, Qiagen, USA). Results were analyzed as per the manufacturer's protocol.

Autophagy Assay

Autophagy was determined using Cyto-ID® Autophagy detection kit (Cat No. ENZ-51031-0050, Enzo Life Sciences, USA). It is based on the ability of Cationic Amphiphilic Tracer (CAT) dye to

accumulate in the vacuoles associated with the autophagy pathway. This can be visualized using either fluorescence microscopy or FACS analysis. HaCaT or NOKSI cells were treated with different laser doses and assay was performed after 24 hrs of treatment. Cells were washed with assay buffer and incubated with dual detection reagent (For every 1 ml of assay buffer, 2 µl of Cyto-ID™ Green Detection Reagent and 1 µl of Hoechst 33342 dye) for 30 minutes at 37 °C. Cells were washed with 100 µl of assay buffer and analyzed by EVOS fluorescence microscopy (Life Technologies, USA) using a standard FITC / DAPI filter. For FACS analysis, incubations were performed with Cyto-ID™ Green Detection Reagent without Hoechst 33342 and fluorescence was assessed (FITC channel 488 nm). FACS analysis using CAT dye was only performed with sub-phototoxic laser doses as this dye did not appear to be incorporated in dead cells.

Immunohistochemistry (IHC)

All the tissue sections were examined by H&E staining for laser damage. Immunostaining for ATF4 and HSP70 were performed as previously described[2]. Briefly, tissues were deparaffinised in xylene for 15 minutes and then transferred to absolute ethanol for 10 minutes followed by incubation in PBS (pH 7.2) for 5 minutes. Deparaffinized sections were subjected for antigen retrieval by boiling in 10 mM citrate buffer (pH 6.0) (MACRON fine chemicals, USA) using a microwave oven for 3 minutes at 850 W. Sections were allowed to cool at room temperature, washed twice in PBS for 5 minutes each and endogenous peroxidase activity was blocked using 5% hydrogen peroxide in methanol. Following a brief PBS wash, blocking was performed with skimmed milk powder (5%) (Carnation, Lab Resources, USA) for 1hr and incubated over night at 4 °C with primary antibodies ATF-4 (1181-5S, Cell Signaling Technology, 1:100), HSP70 (4872P, Cell Signaling Technology, 1:100) and γ-H2AX (9718s, P-Histone H2AX, Cell Signaling Technology, 1:250). The following day, a horseradish peroxidase detection system (QD440-XAK, Biogenex, USA) was used with DAB (Sigma-Aldrich, USA) as a chromogenic substrate to visualize staining. Immunostaining was

quantitated using Image J software (NIH, USA). For quantitation, images were converted to RGB 8 bit images montage and intensity was adjusted to highlight stained areas. Damaged areas assessed are circled in the figures and are compared to surrounding undamaged area, presented as Integrated Density Value (IDV).

Immunocytochemistry

Immunocytochemistry was performed for the detection of γ-H2AX level after treatment with phototoxic laser doses. 15000 HaCaT cells were grown on chamber slide in 0.5 ml of culture medium for 24 hrs. The cells were treated with phototoxic dose of laser but were fixed and permeabilized after 6 hrs of treatment. Cells were fixed and permeabilized in chilled methanol for 10 min at -20° C. Following this, methanol was removed and was washed with phosphate-buffered saline, pH 7.2. Cells were blocked in 1% BSA for 1 hrs. BSA was removed and cells were washed in phosphate-buffered saline. The cells were incubated with γ-H2AX primary antibody for overnight (γ-H2AX, 9718s, P-Histone H2AX, Cell Signaling Technology, 1:250) at 4° C and following day incubated with secondary antibody (Anti-rabbit Alexa Fluor 488) for 1h. Finally, cells were stained with Rhodamine phalloidin (Life Technologies, l:250) for 5-10 min. Cells were visualized with EVOS® FL Cell Imaging System.

ATF-4 Knockdown and Over expression

ATF-4 expression was knockdown in vitro using short interfering RNA (siRNA) approach (Cat. No. 1027416, Qiagen, USA). Target sequence used for ATF-4 knock down is CAGCGTTGCTGTAACCGACAA (SI03019345) along with an AllStars negative control siRNA CAGGGTATCGACGATTACAAA. The siRNAs were reconstituted in sterile water and transfections were performed using HiPerFect transfection reagent (Qiagen, USA), as per the manufacturer's protocol. ATF-4 over expression was carried out using human cDNA clone of ATF-4 (SC322421, Origene, USA).

Western blot analysis

Cells were washed in DPBS (Gibco, USA) and lysed with RIPA lysis buffer containing protease inhibitor cocktail (both, Sigma Aldrich, USA). Total protein was determined using the BCA reagent (Pierce, USA). Equal amount of protein extracted from cells were resolved on 2-10% gradient SDS-PAGE gel, transferred to polyvinylidene difluoride (PVDF) membrane and subjected to immunoblot analysis. To block nonspecific binding sites, blots were incubated in blocking buffer (Licor, USA) for 1 hr followed by overnight incubation in primary antibodies [ATF-4 (ab1371, Abcam), HSP70 (4872P, Cell Signaling Technology), LC3A/B (13118S, Cell Signaling Technology), Cleaved Caspase 3 (9664S, Cell Signaling Technology), eIF2α (5324S, Cell Signaling Technology), Bip (3177S, Cell Signaling Technology) and β-Actin (Cell Signaling Technology)] at 4 °C diluted according to the manufacturer's instructions (dilution 1:1000 in 1% BSA). Blots were washed in Tris-buffered saline with Tween (TBST) thrice (10 min each) followed by incubation with secondary antibody (Anti rabbit/ mouse or goat IRDye 800 or 680 CW, Licor, USA, dilution 1:10000) for 1 hr. Following TBST washes, bands were visualized using two channel Odyssey Imaging Systems (Licor, USA) and quantitated using AlphaImager software (ProteinSimple, USA). Quantitations are presented compared to control (β-Actin). For Cleave Caspase-3, cleaved band was quantified. For LC3A/B, ratios of LC3A/B-II to LC3A/B-I are presented.

Statistical Analyses

Statistical analyses were performed in Graphpad prism software (GraphPad Software, Inc., USA). Significance among two groups was assessed using paired Student's t-test while multiple groups were assessed using analysis of variance (ANOVA) with Bonferroni's Multiple Comparison Test. All treatments were compared to untreated control and $p<0.05$ was considered significant. P value are indicated in figures as < 0.01 (*), < 0.001 (**), < 0.0001 (***), < 0.0001 (****) and not significant (n.s.) respectively.

SUPPLEMENTARY FIGURE LEGENDS

Supplementary Figure 1: Minimal *in vivo* parameters for laser phototoxicity

Images of mice skin treated with laser and maintained at 45 °C for 10 s (a), 45 °C for 30 s (b) and 55 °C for 30 s (c) on day 0 and day 1. Damaged area is circled. Surface temperature profile of mice treated with laser at 45 °C for 10 s (n=6) (d), 45 °C for 30 s (n=13) (e) and 55 °C for 30 s (n=6) (f) as assessed with IR camera. Representative H&E stained sections demonstrating coagulation and necrosis of skin and connective tissue (g), muscle (h) bone marrow (i), peripheral nerve (j) and degenerative changes in spinal cord (k and l) following lethal (> 55 ^{o}C) laser treatments. Insets show higher magnification. Damaged areas indicated by arrows. Scale bar = 70 µm.

Supplementary Figure 2: **Effect of melanin score on laser phototoxicity** (a) Images of mice skin with varying color and melanin score (MS) are shown that were obtained with a Derma lab probe. (b) Average melanin scores of dark (n=39) and pale (n=47) skin colored mice are plotted. Significance was assessed with student's *t*-test. (c) Laser power (Watt) needed to attain skin surface temperature of 45 °C is plotted for pale and dark skin mice (n=3). (d) Time needed to reach the skin surface temperature of 45 °C is plotted for pale and dark skin coloured mice. Significance based on student's *t*-test compared (n=5). Representative thermal profiles of dark (e) and pale (f) color skin mice are plotted against time prior to reaching the phototoxic temperature at 45 °C. (g) Metal probe heated at 45 °C were placed firmly on mice skin for 30 s and skin damage was monitored for 3 days. (h) Absorption of 810 nm laser at 2 W/cm^2 through different layers of mice or through black and clear tissue culture plates were measured using power meter. (i) Laser treatments were performed in clear and black well plates and cellular viability was assessed 24 hrs after treatment with AlamarBlue and a color change is shown. Statistical significance are indicated as P < 0.001 (**) and <0.00001 (****).

Supplementary Figure 3: **Effects of absorption area, mass and pre-treatment temperature on laser phototoxicity** (a) Laser treatments (phototoxic dose in 96 well plate) on HaCaT and NOKSI cells were performed in black well plates with clear bottom covered with rubber mastic tape over various surface areas and cellular viability was assessed at 24 hrs after treatment (n=3). (b) Surface temperature of cells treated with laser as assessed by IR camera (n=3) is shown. (c) HaCaT and NOKSI cells were pre-incubated at 4 ^{o}C for 15 min and then laser treated with phototoxic doses and were assessed for cell viability at 24 hours (n=3). (d) Cells were pre-incubated at 42 °C and laser treatments were performed with varying doses and cell viability was assessed at 24 hrs (n=3). Laser phototoxicity was also assessed with varying volumes of media (n=3) (e) as well as varying specific heat of culture media during treatments (10% DMEM versus PBS) (n=3) (f) and cell viability was assessed at 24 hrs. (g) HaCaT cells were treated with phototoxic dose followed by TUNEL assay to detect apoptosis. Scale bars = 70 μm. Enzyme activities of purified Glutathione Reductase (GR) (h) and Catalase enzymes (i) were assessed following phototoxic laser dose in black well plates (n=3). (j) *In vitro* ratio of GSSG/GSH were assessed in HaCaT and NOKSI cells treated with phototoxic laser doses (n=3). (k) During ROS neutralization experiment to assess its effect at phototoxic doses of laser treatments on cellular viability, rise in surface temperature was measured using IR camera. Statistical significance was determined using two-way analysis of variance (ANOVA) among different treatments using the Bonferroni's multiple comparison test. Statistical significance are indicated as $P < 0.01$ (*), 0.001 (**), < 0.0001(***) and < 0.00001(****).

Supplementary Figure 4: Laser does not cause DNA damage

Quantitation of plasmid cleavage assay performed using PUC19 plasmid treated by laser with varying wavelengths (a) and doses (b) (n=2). Plasmid cleavage assay was analyzed by gel electrophoresis on 1% agarose gel by treating with different wavelengths of broad light sources

(n=3) (c) and LED (Light Emitting Diode) (n=3) (d) at phototoxic dose. Quantitation of the bands by densitometry is shown below (e, f) respectively. (g) NOKSI cells were treated with different doses of laser and genomic DNA was assayed for number of abasic sites. UV treatment was used as a positive control, Significance was based on one-way ANOVA with the respective controls (n=3). (h) Standard curve showing increase in OD with increasing abasic (apurinic assay) sites. Statistical significance was determined using one-way ANOVA among different treatments using the Bonferroni's multiple comparison test. Statistical significance are indicated as $P < 0.0001$(***) and < 0.00001(****). (i) γ-H2AX immunostaining on mice lungs were performed to assess DNA damage with γ-radiation (10 Gy) as a positive control.

Supplementary Figure 5: **Non-supervised hierarchical clustering and status of ER stress pathway**

(a) Differentially induced genes in radiation-treated HaCaT cells were compared to laser-treated group by using a non-supervised hierarchical clustering. Highlighted genes (horizontal black color box) are part of ER stress pathway. Majority of the differentially regulated genes in radiation are not regulated in the laser treated group (highlighted vertically in gray color box). (b) Status of ER stress marker IRE1α was assessed using immunoblotting following laser treatment with different doses. (c) Laser treatment of HaCaT cells with phototoxic dose in black well plates and cleavage of XBP1 was analyzed using semi-quantitative PCR and normalized to RPL35A expression. γ-radiation was used as a positive control.

Supplementary Figure 6: Laser induces ER stress at sub-phototoxic dose while suppresses it at phototoxic dose

Laser treatment was performed at increasing doses in HaCaT (a) and NOKSI (b) cells in clear and black well plates and evaluated for ER stress markers by immunoblotting. β-Actin was used as

normalizing control. Autophagy was assessed with CAT dye (Cyto-ID® Autophagy detection kit) by fluorescence imaging (c) and FACS analysis (n=3) (d) following laser treatments of HaCaT and NOKSI cells. (e) Pretreatment with an autophagy inhibitor (3-MA; 3-Methyladenine) was subjected to sub-phototoxic laser doses and autophagy was quantitated using the CAT dye by fluorescence imaging. Scale bars = 200 µm. Statistical significance was determined using two-way analysis of variance (ANOVA) with Bonferroni's multiple comparison test. Phototoxic dose of laser is highlighted in gray font. Statistical significance is indicated as *P* < 0.00001(****).

Supplementary Figure 7: ATF-4 knock down cells are sensitive while stable over expressing clones are resistant to laser phototoxicity

(a) HaCaT and NOKSI cells were transfected with ATF-4 siRNA in two different ratio (siRNA: Hiperfect) and % knock down were assessed using Real time PCR (n=3). (b) ATF-4 knock down NOKSI cells were treated with increasing doses of laser and cell viability was assessed at 24 hrs (n=3). (c) HaCaT cells were transfected with ATF-4 siRNA and were treated with different laser doses for 24 hrs and were assessed by immunoblotting for ER stress pathway. Real time PCR quantitation of ATF-4 overexpressing stable clones of HaCaT (d) and NOKSI (e) cells. Clone 4 and Clone 6 of HaCaT and Clone 1 and Clone 3 of NOKSI were chosen for phototoxicity studies. (f) NOKSI stable cells over expressing ATF-4 were treated with increasing doses of laser and cell viability was assessed (n=3). Statistical significance was determined using two-way analysis of variance (ANOVA) and Bonferroni's multiple comparison test. Statistical significance are indicated as *P* < 0.05 (*), <0.001 (**), <0.0001 (***), <0.00001(****) and not significant (n.s.). (g) Histological assessment of mice skin with H&E showing damaged area in laser-treated skin.

Supplementary Figure 8: Laser phototoxic treatment suppresses ATF-4 and HSP70 while it gets retained if ROS or temperature is neutralized.

(a) The dorsal skin of 5 week old C57BL/6NCr male mice were subjected to laser treatments to maintain different temperatures and the tissue was collected at 24 hrs post-treatment and assessed by immunoblotting for ATF-4 and HSP70 levels. Normalization with β-Actin is shown. (b) Phototoxic laser treatments were performed on NOKSI cells with or without cooling and ROS induction was assessed with DCFDA using FACS analyses. (c) Laser treated mice skin tissues in ROS neutralization experiment with NAC, NAA or cooling were assessed by immunostaining for ATF-4 and HSP70 expression (Scale bars = 70 µm.). (d) Compromise of ER stress (markers) in HaCaT cells at 4 °C, compared to 37 °C, following laser treatments. Statistical significance was determined using one-way analysis of variance (ANOVA) and Bonferroni's multiple comparison test. Statistical significance is indicated as $P < 0.05$ (*).

Supplementary Video 1: Phototoxic laser damage at high temperatures (> 55 ^{o}C) demonstrating involuntary lower limb extension (leg rise). Inset demonstrates surface temperature monitored with infrared camera at concurrent time points. Final still image shows raised leg with the laser probe placed as reference on table top immediately post-treatment.

References

1. Basu, U., *et al.* Nuclear targeting terpyridine iron(II) complexes for cellular imaging and remarkable photocytotoxicity. *J Inorg Biochem* **116**, 77-87 (2012).
2. Khan, I., *et al.* Role of TGF-beta and BMP7 in the pathogenesis of oral submucous fibrosis. *Growth Factors* **29**, 119-127 (2011).
